# TSPO acts as an immune resistance gene involved in the T cell mediated immune control of glioblastoma

**DOI:** 10.1186/s40478-023-01550-9

**Published:** 2023-05-08

**Authors:** Ayse N. Menevse, Laura-Marie Ammer, Arabel Vollmann-Zwerenz, Marcell Kupczyk, Julia Lorenz, Lorraine Weidner, Abir Hussein, Julian Sax, Jasmin Mühlbauer, Nicole Heuschneider, Celine Rohrmus, Laura S. Mai, Birgit Jachnik, Slava Stamova, Valentina Volpin, Franziska C. Durst, Antonio Sorrentino, Maria Xydia, Vladimir M. Milenkovic, Stefanie Bader, Frank K. Braun, Christian Wetzel, Nathalie L. Albert, Joerg-Christian Tonn, Peter Bartenstein, Martin Proescholdt, Nils O. Schmidt, Ralf A. Linker, Markus J. Riemenschneider, Philipp Beckhove, Peter Hau

**Affiliations:** 1 Division of Interventional Immunology, Leibniz Institute for Immunotherapy (LIT), 93053 Regensburg, Germany; 2 Wilhelm Sander-NeuroOncology Unit and Department of Neurology, University Hospital Regensburg, 93053 Regensburg, Germany; 3 Department of Neuropathology, University Hospital Regensburg, 93053 Regensburg, Germany; 4 Department of Psychiatry and Psychotherapy, University of Regensburg, Molecular Neurosciences, 93053 Regensburg, Germany; 5 Department of Nuclear Medicine, University Hospital of Munich, LMU Munich, 80336 Munich, Germany; 6 Department of Neurosurgery, University Hospital of Munich, LMU Munich, 80336 Munich, Germany; 7 Department of Neurosurgery, University Hospital Regensburg, 93053 Regensburg, Germany; 8 Department of Neurology, University Hospital Regensburg, 93053 Regensburg, Germany; 9 Department of Internal Medicine III, University Hospital Regensburg, 93053 Regensburg, Germany; 10 Department of Neurology –NeuroOncology, University Hospital Regensburg, Franz-Josef-Strauß-Allee 11, 93053 Regensburg, Germany; 11 LIT – Leibniz Institute for Immunotherapy (former RCI), c/o Universitätsklinikum Regensburg, Franz-Josef-Strauß-Allee 11, 93053 Regensburg, Germany

**Keywords:** TSPO, GB, Anti-tumor immunity, Immune-resistance, TRAIL-resistance

## Abstract

Glioblastoma (GB) IDH-wildtype is the most malignant primary brain tumor. It is particularly resistant to current immunotherapies. Translocator protein 18 kDa (TSPO) is upregulated in GB and correlates with malignancy and poor prognosis, but also with increased immune infiltration. Here, we studied the role of TSPO in the regulation of immune resistance of human GB cells. The role of TSPO in tumor immune resistance was experimentally determined in primary brain tumor initiating cells (BTICs) and cell lines through genetic manipulation of TSPO expression and subsequent cocultures with antigen specific cytotoxic T cells and autologous tumor-infiltrating T cells. Death inducing intrinsic and extrinsic apoptotic pathways affected by TSPO were investigated. TSPO-regulated genes mediating apoptosis resistance in BTICs were identified through gene expression analysis and subsequent functional analyses. TSPO transcription in primary GB cells correlated with CD8^+^ T cell infiltration, cytotoxic activity of T cell infiltrate, expression of TNFR and IFNGR and with the activity of their downstream signalling pathways, as well as with the expression of TRAIL receptors. Coculture of BTICs with tumor reactive cytotoxic T cells or with T cell-derived factors induced TSPO up-regulation through T cell derived TNFα and IFNγ. Silencing of TSPO sensitized BTICs against T cell-mediated cytotoxicity. TSPO selectively protected BTICs against TRAIL-induced apoptosis by regulating apoptosis pathways. TSPO also regulated the expression of multiple genes associated with resistance against apoptosis. We conclude that TSPO expression in GB is induced through T cell-derived cytokines TNFα and IFNγ and that TSPO expression protects GB cells against cytotoxic T cell attack through TRAIL. Our data thereby provide an indication that therapeutic targeting of TSPO may be a suitable approach to sensitize GB to immune cell-mediated cytotoxicity by circumventing tumor intrinsic TRAIL resistance.

**Supplementary Information:**

The online version contains supplementary material available at 10.1186/s40478-023-01550-9.

## Introduction

Glioblastoma isocitrate dehydrogenase (GB IDH)-wildtype (CNS WHO grade 4) is the most common malignant type of primary brain tumor. Despite maximal possible resection, radiotherapy, chemotherapy with the alkylating agent temozolomide and treatment with tumor treating fields, tumors nearly always relapse, leading to a median survival of only 16 to 20.9 months [68, 79, 86, 87].

Inferior survival times and unsatisfying treatment results emerge, at least in part, from the immense inter- and intratumoral heterogeneous nature of GB [5, 23]. To better understand this complexity, GB was classified into molecular subtypes that relate to clinical features and prognosis, based on transcriptomic profiling [7, 92]. Single-cell RNA sequencing (RNA-Seq) analysis of GB demonstrated that a combination of single cells representing multiple subtypes can co-exist within the same tumor [65, 73]. The presence of brain tumor-initiating cells (BTICs) with stem-cell-like characteristics adds another level of complexity and contributes to the therapy resistance of GB [24, 61, 81].

Besides molecular alterations, the pathogenesis of GB is also dependent on immune escape mechanisms developed by the tumor. Although high infiltration of cytotoxic CD8^+^ T cells is associated with prolonged survival, tumor cells inhibit T cell function by secreting immunosuppressive factors such as TGF-β and IDO [43, 53, 60]. Also an up-regulation of co-inhibitory immune checkpoint molecules such as Programmed Cell Death 1 ligand 1 (PD-L1) may contribute to immune suppression in some cases, although different studies on PD-L1 expression and its` relevance in GBM immune surveillance revealed inconsistent results [6, 7, 21, 31]. Therapeutic blockade of a single immune checkpoint target may lead to usage of alternative immune checkpoints and genes that confer increased resistance against the cytotoxic attack by immune cells [16, 45, 83, 94]. So far, neoadjuvant anti-PD-1 therapy could not improve clinical outcomes in GB, even though it can elevate Interferon gamma (IFNγ)-related T cell responses. This points to an induction of adaptive immune resistance mechanisms [18]. The notorious ineffectiveness of so far developed immunotherapeutic strategies for GB also suggests that additional tumor intrinsic immune resistance mechanisms exist that are still unknown.

One gene whose expression is associated with the particularly immunogenic mesenchymal GB subtype is Translocator protein (18 kDa) (TSPO) [15, 21]. TSPO is a ubiquitous mitochondrial protein that is particularly abundant in steroid producing tissues. TSPO is overexpressed in GB compared to non-neoplastic brain tissue and its expression correlates with poorer prognosis and survival of patients. More specifically, TSPO expression is higher in IDH wildtype compared to IDH-mutant gliomas (WHO grade 4). Within the IDH-mutant tumors, TSPO expression positively correlates with WHO grade [15, 21]. TSPO plays a role in steroid synthesis, regulation of mitochondrial respiration and oxidative stress and its expression has been linked to increased tumor cell proliferation, invasion and overall resistance to apoptosis [2, 9, 59, 75, 102, 103]. In addition to that, its expression is associated with increased inflammatory activity in the brain [66]. However, a role of TSPO in anti-tumor immune response in GB remains unknown so far.

Here, we report that cytotoxic T cells trigger TSPO upregulation in GB cells through the release of inflammatory cytokines and that TSPO mediates immune resistance of GB cells against a cytotoxic T cell attack through suppressing death receptor ligand Tumor necrosis factor-related apoptosis-inducing ligand (TRAIL) mediated apoptosis.

## Materials and methods

### Ethics

All procedures performed in studies involving human participants were in accordance with the ethical standards of the institutional research committee and with the 1964 Helsinki Declaration and its later amendments or comparable ethical standards. The sampling of tumor specimens and enrichment of BTICs was approved by the Ethics Committee of the University of Regensburg (No° 18–207-101, renewed in 05/2022). The sampling of tumor infiltrating lymphocytes (TILs) was approved by the Ethics Committee of the University of Regensburg (No° 21–2393-1–101). Informed consent was obtained from all individual participants included in the study.

### Cell culture and lentiviral transduction

BTIC13, -111, -129 and -134 brain-tumor-initiating cells (BTIC) were established from resections of human GB (CNS-WHO-grade 4) as previously described [47]. Cell lines were authenticated via STR profiling by a commercial provider (eurofins Genomics). Tumor cells were maintained in RHB-A-based serum-free culture media (Takara, #Y40001), supplemented with Penicillin–Streptomycin (Merck, #P4333), 20 ng/ml each of epidermal growth factor (EGF; Miltenyi Biotec, #130–097-751) and basic fibroblast growth factor (bFGF; Miltenyi Biotec, #130-093-842). Cells were incubated at 37 °C, 5% CO_2_ and 95% humidity.

To achieve stable knockdown of TSPO, BTIC13, -111, -129 and -134 were lentivirally transduced. TSPO-specific short hairpin RNA (shRNA) sequence (shTSPO-F: 5’-CCG GCC ACA CTC AAC TAC TGC GTA TCT CGA GAT ACG CAG TAG TTG AGT GTG GTT TTT G-3’, shTSPO-R: 5’-AAT TCA AAA ACC ACA CTC AAC TAC TGC GTA TCT CGA GAT ACG CAG TAG TTG AGT GTG G-3’) and a non-targeting control shRNA were cloned into pLKO.1 puro plasmids (gift from Bob Weinberg; Addgene plasmid #8453) [59]. To generate lentiviral particles, shRNA plasmids were co-transfected with pCMV-VSV-G and psPAX2 (kindly gifted by Prof. S. Geley, University of Innsbruck, Austria) in human embryonic kidney (HEK) 293-T cells using Metafectene (Biontex; #T020-1.0). After 24 h, filtrated supernatants from HEK cells containing lentiviral particles were used to transduce BTIC target cells by spinoculation (900 rpm, 1 h, room temperature). Transduced cells were selected by treatment with Puromycin (Merck, #P8833; 3 µg/ml) 24 h after transduction.

Clonal cell lines of BTIC13 TSPO knockdown and control cells were obtained by single cell dilution. Isolated single cells were seeded in 96 well plates and sub-cultured stepwise into larger vessels until they could be used for experiments.

Human GB cell lines U-87 MG (ATCC®, HTB-14™; source: male) and U-251 MG (ECACC, 09,063,001; source: male) were kindly provided by Prof. Markus J. Riemenschneider. Cells were cultured under standard conditions in DMEM (Merck, #D6429) supplemented with 10% fetal calf serum, 100 U/ml penicillin G and 100 µg/ml streptomycin at 37 °C in a humidified atmosphere under 5% CO2. Human glial (oligodendrocytic) hybrid cells (MO3.13; Tebubio, CLU301-P, source: female) were cultured in high glucose DMEM without sodium pyruvate (Merck, #D5796) supplemented with 10% fetal calf serum, 100 U/ml penicillin G and 100 µg/ml streptomycin at 37 °C in a humidified atmosphere under 5% CO2.

### Generation of tumor-infiltrating lymphocytes (TILs)

Freshly resected GB tissue was cut in small pieces and enzymatically digested in control media supplemented with 400 µg/ml DNase I (Merck, #11284932001) and 400 U/ml collagenase type IV (Worthington, #LS004209) for 2 h at 37 °C, 5% CO_2_. The digested tumor was then filtered through a 100 µm filter, centrifuged at 1400 rpm for 10 min and co-cultured with 2 × 10^7^ irradiated allogeneic PBMCs from 3 different donors in 20 ml of expansion medium supplemented with 30 ng/ml anti-CD3, 3000 U/ml IL-2 and 0,38 µg/ml Amphotericin B (Gibco, #15290–26) for 14 days. On days 5, 7 and 11, IL-2 was replenished with fresh expansion medium. On day 14, cells were collected, characterized by FACS and frozen in aliquots of 5 × 10^6^ in freezing media A (60% AB serum and 40% RPMI1640) and B (80% AB serum and 20% DMSO).

### Generation of flu-antigen specific CD8^+^ T cells (FluTC)

Flu antigen-specific T cells (FluTC) were generated as previously described [94]. Briefly, peripheral blood mononuclear cells (PBMCs) were isolated from buffy coats of HLA-A2^+^ healthy donors by density gradient centrifugation (Biocoll, Biochrom, # L6715). On day 0, total CD8^+^ T cells were sorted from PBMCs by magnetic separation (CD8^+^ T Cell Isolation Kit, Miltenyi, #130–096-495) according to the manufacturer’s instructions and expanded in the presence of HLA-A2-matched Influenza (flu) peptide (GILGFVFTL, ProImmune, #P007-0A-E) for 14 days. The autologous CD8 negative fraction was irradiated with 60 Gray (IBL 437C Blood Irradiator) and used for 1 week as feeder cells which were then substituted by irradiated T2 cells. On day 1 and day 8, 100 U/ml IL-2 (Clinigen, PZN-16771811) and 5 ng/µL IL-15 (R&D Systems, #247-ILB/CF) were added to the expansion. The percentage of FluTC was determined by pentamer staining (GILGFVFTL-APC, ProImmune #F007-4A-E) on day 7 and day 14 via flow cytometry (FACS). After antigen-specific expansion, 1 × 10^6^ FluTC were sorted by FACS and expanded further for 14 days by using a modified version of the Rosenberg protocol [22]. Briefly, on day 0, sorted FluTC were cultured in 150 ml expansion media (50% AIM-V (Thermo Fisher Scientific, #12055091), 50% CLM (CLM: RPMI1640 (Thermo Fisher Scientific, #21875091), 10% human AB serum (Valley Biomedical, #HP1022HI), 1% HEPES (Merck, #H0887), 100 U/ml penicillin G, 100 µg/ml streptomycin and 0.01% Beta-mercaptoethanol (Thermo Fisher Scientific, #31350–010) supplemented with 30 ng/ml anti-CD3 (Clone: OKT3, eBioscience, #14–0037-82), 3000 U/ml IL-2 and 2 × 10^8^ irradiated allogeneic PBMCs from 3 different healthy donors. On days 5, 7 and 11, IL-2 was replenished with fresh expansion media. On day 14, cells were collected, characterized by FACS and frozen in aliquots of 5 × 10^6^ in freezing media A (60% AB serum and 40% RPMI1640) and B (80% AB serum and 20% DMSO (Merck, #D260)). On the day of usage, FluTC were thawed at least 4 h prior to the co-culture or activation and cultured in CLM at a concentration of 1 × 10^6^ cells/ml.

### Generation of supernatants of polyclonally activated T cells

Non-tissue culture 6-well plates were coated with 4 µg/ml anti-CD3 antibody (Clone: OKT3, eBioscience, #14–0037-82) overnight at 4 °C. On the next day, wells were washed two times with 1 ml PBS and FluTC/TILs were seeded in CLM supplemented with 1 µg/ml of human anti-CD28 antibody (Clone: CD28.2, BioLegend, #302902) at a concentration of 1 × 10^6^ cells/ml. After 24 h activation, cell suspension was centrifuged at 1400 rpm for 10 min and activated supernatant was collected into a fresh tube for further use. Activated TIL129 were resuspended in CLM and further used for co-culture assays.

### Reverse small interfering RNA transfection

U-87 MG and U-251 MG cells were transfected either with non-targeting control small interfering RNA (siRNA) (Horizon, D-001810–04) or a pool of 4 TSPO specific siRNAs (Sequences: 5’-ACA CUC AAC UAC UGC GUA U-3’; 5’-CUU CUU UGG UGC CCG ACA A-3’; 5’-GGG CCU UGG UGG AUC UCC U-3’; 5’-CUU GUG AUG UGG UGG CCG U-3’, Horizon, MQ-009559–03) using RNAiMAX (Thermo Fisher Scientific, #13778–150) according to manufacturer`s instructions. Briefly, 10 µL of 250 nM siRNA solution was added to each well of a 96-well plate. 0.1 µl of RNAiMAX transfection reagent was diluted in 10 µL of RPMI (Merck, #R8758) and incubated for 10 min at room temperature. 20 µL of RPMI was added and 30 µL of RNAiMAX mix was given to the wells coated with siRNA and incubated for 30 min at room temperature. 5 × 10^3^ U-87 MG or U-251 MG cells were resuspended in 60 µL DMEM containing 10% FCS, seeded in the siRNA-RNAiMAX containing wells and incubated for 72 h at 37 °C, 5% CO2. For 6-well plate transfection, the abovementioned protocol was proportionally scaled up.

### RNA Isolation, Reverse Transcription and quantitative RT-PCR

Total RNA was extracted from cultured cells using RNeasy Mini kit (Qiagen, #74106) and 500–1000 ng of RNA were transcribed using the QuantiTect reverse transcription kit (Qiagen, #205313) according to the manufacturer's protocol. For real-time quantitative PCR (RT-qPCR) 10 ng of template cDNA, 2x QuantiFast SYBR Green PCR mix (Qiagen, #204056) and 300 nM of gene-specific primer mix (TSPO fw: TCT TTG GTG CCC GAC AAA T; TSPO rev: GGT ACC AGG CCA CGG TAG T, Merck; PD-L1 fw TGC CGA CTA CAA GCG AAT TAC TG, PD-L1 rev: CTG CTT GTC CAG ATG ACT TCG G, Merck; RT^2^ qPCR Primer Assay for Human PI3, Qiagen, PPH11211A-200; RT^2^ qPCR Primer Assay for Human SLPI, Qiagen, PPH02863A-200) were used per 20 μL reaction and each sample was prepared in triplicates. Reactions were run using the QuantStudio 3 (Thermo Fisher Scientific). Expression of the target genes was normalized to the expression of the reference gene (β-actin fw: CCT CGC CTT TGC CGA TCC, β-actin rev: GCG CGG CGA TAT CAT CAT CC; RPL13A fw: CAT AGG AAG CTG GGA GCA AG, RPL13A rev: GCC CTC CAA TCA GTC TTC TG; or GAPDH fw: AGC CAC ATC GCT CAG ACA C, GAPDH rev: AAT ACG ACC AAA TCC GTT GAC T) and the analysis was performed using comparative Ct method (ΔΔCT). The results were shown as fold change compared to the control sample.

### Next-generation RNA-sequencing

Libraries for next-generation sequencing (NGS) were prepared from 200 ng total RNA with the Illumina Stranded mRNA Prep kit (Illumina, #20040534) according to manufacturer`s instructions. NGS was performed on a NextSeq 550 Dx instrument using indexed, 150 cycles paired end read protocol and the NextSeq 500/550 High Output Kit v2.5 (Illumina, #20024907). Analysis of NGS data was performed using only freely available, customizable tools and a workstation. Image analysis and base calling resulted in.bcl files which were converted into.fastq files by the bcl2fastq2 tool v2.17.1.14. Next,.fastq files were mapped to the human genome assembly GRCh38.87 using the HiSat2 Mapper allowing one mismatch [42]. All unique hits were further processed using featureCounts v2.0.1 to create count tables for all samples [51]. Reads were counted locus-based, i.e. for unions of exons of genes. Batch effects were removed with Combat_Seq of the SVA package using a negative binomial regression model that retains the integer nature of count data in RNA-seq studies [105]. PCA and differential expression analysis were generated with R v4.1.2 using DeSeq2 [54]. Volcano plots and heatmaps were generated with the Enhanced Volcano R package and Interactive Complex Heatmap R package [8, 28].

### ELISA and Luminex assay

Supernatants of T cell-tumor cell co-cultures and anti-CD3/CD28 activated T cells were analyzed for the detection of IFNγ (Human IFN-γ ELISA Set, BD OptEIA #555142), Tumor necrosis factor alpha (TNFα) (Human TNF ELISA Set, BD OptEIA #555212), Granzyme B (Human Granzyme B ELISA development kit, Mabtech #3485-1H-20) and TRAIL (human TRAIL/TNFSF10 DuoSet ELISA, R&D Systems, #DY375-05). Experiments were performed according to the manufacturer´s instructions. Absorbance was measured at λ = 450 nm, taking λ = 570 nm as reference wavelength using the Spark 10 M multimode microplate reader (TECAN). Soluble Fas ligand (FasL) was detected by multiplex cytokine assay (MILLIPLEX Human CD8^+^ T Cell MAGNETIC Premixed 17 Plex Kit, Merck, #HCD8MAG15K17PMX). The assay was performed according to the manufacturer´s instructions and samples were measured using the MAGPIX Luminex instrument (Merck).

### Western blot analysis

Western blot was performed as described [47]. Briefly, cell pellets were lysed in RIPA buffer for 10 min on ice and centrifuged for 10 min (14,000 rpm, 4 °C). Protein concentration was determined using BC Assay Protein Quantification kit (Interchim, #UP40840A). Equal amounts of protein were diluted in 5x Laemmli buffer, boiled, separated on 12% SDS PAGE and transferred to PVDF membranes (VWR, #516–0224) which were blocked in 5% nonfat dry milk in TBS-T buffer. Incubation with indicated primary antibodies was performed overnight at 4 °C. Afterwards, membranes were washed with TBS-T buffer and incubated with horseradish peroxidase (HRP)-conjugated secondary antibodies. Detection was performed using the Immobilon Forte Western HRP Substrate (Millipore, #WBLUF0100). Protein detection was performed with ImageQuant TL software (GE Healthcare, TL 8.1). Relative protein expression was determined by normalizing the protein of interest to the loading control as well as to the treatment control. The following primary antibodies were used: rabbit anti-Caspase 9 (Cell signaling Technologies, #9502, 1:1000), rabbit anti-Caspase 3 (Cell signaling Technologies, #9662, 1:1000), rabbit anti-cleaved Caspase 3 (Cell signaling Technologies, #9664, 1:1000), rabbit anti-PARP1 (Cell signaling Technologies, #9532, 1:1000), rabbit anti-TSPO (kind gift from V. Milenkovic, 1:5000), mouse anti-GAPDH (Santa Cruz, #47724, 1:2500) and mouse anti-ß-Actin (Merck, #A5316, 1:2500). The HRP-labeled anti-mouse IgGκ binding protein (Santa Cruz, #sc516102, 1:5000) and the mouse anti-rabbit IgG antibody (Santa Cruz, #sc-2357, 1:5000) were used for detection of primary antibodies.

### Flow cytometry

Flow cytometry was used for the detection of proteins expressed on the plasma membrane of tumor and T cells as published [83]. Tumor cells were detached from plates using PBS-EDTA, centrifuged at 500 × g for 5 min and resuspended in FACS buffer (2 × 10^5^ cells/tube). Live cells were distinguished by using Zombie Aqua™ (BioLegend, #423102) or Zombie NIR™ Fixable Viability Kit (#423106) according to manufacturer’s instructions followed by blocking with Kiovig (human plasma-derived immunoglobulin, Baxter, PZN- 06587176) at a concentration of 100 µg/mL in FACS buffer (PBS, 2% FCS) for 15 min in the dark on ice. Tumor cells were washed once in FACS buffer and incubated for 20 min on ice in the dark with either fluorophore-conjugated primary antibodies or isotype control: PE anti-CD119 (IFN-γ R α chain) (Clone: GIR-208, BioLegend, #308606); APC anti-CD120a (TNF-R1) (Clone: W15099A, BioLegend, #369906); PE anti-CD120b (TNF-R2) (Clone: 3G7A02, BioLegend, #358404); BV421 anti-CD95 (Fas) (Clone: DX2, BioLegend, #305624); APC anti-CD261 (DR4, TRAIL-R1) (Clone: DJR1, BioLegend, #307208); PE anti-CD262 (DR5, TRAIL-R2) (Clone: DJR2-4 (7–8), BioLegend, #3007406); anti-human HLA-A,B,C (MHC-I) (Clone: W6/32, BioLegend, #311402). For the MHC-I staining, cells were washed once and incubated with the secondary antibody BV605 anti-Mouse IgG2a/IgG2b (Clone: R2-40, BD, #744294) for another 20 min on ice in the dark. T cells were stained using the same protocol with the following antibodies or their respective isotype controls: AF-700 anti-CD3 (Clone: UCHT1, BioLegend, #300424); PerCP-Cy5.5 anti-CD4 (Clone: RPA-T4, BioLegend, #300530); V450 anti-CD8 (Clone: RPA-T8, BD Bioscience, #560347); APC-Cy7 anti-CD45RO (Clone: UCHL1, BioLegend, #304227); BV605 anti-CD62L (Clone: DREG-56, BioLegend, #304833); APC anti-PD-1 (Clone: EH12.2H7, BioLegend, #329908); FITC anti-LAG3 (Clone: 11C3C65, BioLegend, #369307); PE-Cy7 anti-TIM3 (Clone: F38-2E2, eBioscience-Thermo Fisher Scientific, #25–3109-41); PE anti-TRAIL (Clone: RIK-2, BioLegend, #308206). FluTC were stained with pentamer (A*02:01 GILGFVFTL, Proimmune, #F007-4A-E) prior to antibody staining for 10 min at room temperature in the dark. For IFNγ and TNFα cytokine secretion assay, TIL129 were co-cultured with BTIC129 for 12 h. Afterwards, TILs were stained using IFN-γ (MACS Miltenyi, #130–090-433) and TNF-α Secretion Assay Kits (#130–091-267) according to manufacturer´s instructions. Stained cells were washed once, acquired with the FACSLyric flow cytometer (BD Bioscience) and data were analyzed using FlowJo (Tree Star).

### Immunocytochemistry

BTIC13 wildtype and TSPO knockdown cells were grown on laminin-coated 8-well LabTek Chamber slides (Thermo Scientific, #154534PK) for 24 h. Cells were fixed with 4% paraformaldehyde for 10 min at room temperature and washed three times with 1x PBS. After blocking and permeabilizing with 0.5% Triton X-100 (Merck, #23472–9), 10% donkey serum (Merck; #S30-M) in 1 × PBS for 45 min at room temperature, cells were incubated overnight at 4 °C with rabbit anti-TSPO antibody (Abcam, #ab10949, 1:500) and mouse anti-ATPB antibody (Abcam, #ab14730, 1:1000) in 0.1% Triton X-100, 2% donkey serum in 1x PBS. After three washing steps with 1x PBS, cells were incubated with secondary antibodies donkey anti-rabbit Alexa Fluor 488 (Life Technologies, #A21206, 1:500), donkey anti-mouse Alexa Fluor 568 (Life Technologies, #A10042, 1:1000) and DAPI (Merck, #D9542, 5 µg/ml). After three final washing steps, slides were mounted with ProLong Gold Antifade Mountant (Invitrogen, #P36930) and dried at room temperature. Slides were analyzed with an inverted fluorescence microscope (Zeiss, Axio Observer.Z1) and the VisiView software (Visitron Systems, Version 2.08).

### T-cell mediated TSPO induction

For the analysis of T-cell mediated regulation of TSPO expression in BTICs, 4 × 10^5^ BTIC13, 129 or 131 were seeded in 6-well plates (TPP) in supplemented RHB-A media. After 1 day, cells were pulsed with flu-peptide at the indicated concentrations for 1 h, washed and co-cultured either with 4 × 10^5^ FluTC, or treated with culture supernatant of indicated T cells. BTICs kept in plain CLM were used as control. After 18 h, supernatants of co-cultures were collected for cytokine analysis, T cells were washed away and remaining BTICs were collected. For cytokine treatment, BTICs were cultured for 1 day in CLM and then treated with TNFα (PeproTech, #300-01A) or IFNγ (PeproTech, #300–02) or were left untreated for 24 and 48 h. For cytokine receptor blocking, BTICs were pre-incubated with human anti-TNF-R1 (10 µg/ml, R&D Systems, #MAB625), anti-TNF-R2 (10 µg/ml, R&D Systems, #MAB726), anti-IFNG-R1 (2 µg/ml, R&D Systems, #MAB6731) antibodies or mouse IgG1 isotype control (R&D Systems, #MAB002) in 500 µl CLM for 30 min at 37 °C, 5% CO2. After 30 min incubation, 1.5 ml of indicated T cell culture supernatant was added. After 24 h, cells were pelleted and snap-frozen for further mRNA and protein analysis.

### Cell proliferation and viability assay

Cells were cultured in 96 well black flat bottom plates (SPL Life Sciences, #30296) at a density of 5 × 10^3^ cells / well in 100 µl of growth medium. 24 h later, cells were treated with specified concentrations of TRAIL (PeproTech, #310–04), TNFα (PeproTech, #300-01A), IFNγ (PeproTech, #300–02) or FasL (BioLegend, #589404) for 48 h. 10% of Resazurin solution (R&D Systems, #AR001) was applied 4 h prior to measurement and fluorescence was measured at 560 nm excitation / 590 nm emission with the VarioSkan Flash Multimode Reader (Thermo Fisher Scientific). Assays were performed in quadruplicates and repeated three times. Values were normalized to 0 ng/ml of treatment.

### XTT-assay

Specific T-cell mediated killing of tumor cells was determined by a XTT-based colorimetric cell viability assay as published [36]. After 3 days of siRNA transfection (as described above), U-87 MG and U-251 MG cells were loaded with 0.01 µg/ml flu-peptide (GILGFVFTL, Proimmune, #P007-0A-E). After 1 h of incubation, the pulsing medium was removed and 2 × 10^4^ FluTC or plain CM was added in 100 µl to the wells. shRNA transduced 5 × 10^3^ BTIC13 or 1 × 10^4^ BTIC129 cells were seeded per 96-well in 100 µl fully supplemented RHB-A media 1 d before the co-culture. Next day, BTIC13 cells were loaded with 0.001 µg/ml flu-peptide and co-cultured with 2 × 10^4^ FluTC, whereas BTIC129 were directly co-cultured with 1 × 10^5^ pre-activated autologous TIL129 in 100 µl CM. After 4 h of co-culture, the viability of target cells was measured by adding 50 µl of 1 mg/ml XTT reagent solution (Biomol, #52629500) and 1 µl of 1.25 mM Phenazine Methosulfate (PMS) (Biomol, #Cay30558-5) to each well. Plates were incubated at 37 °C and 5% CO2 and absorption was detected after 30, 60, 90 and 120 min using a Spark 10 M multimode microplate reader (TECAN) (450 nm with 650 nm set as reference). Maximal reduction of XTT by viable cells was determined as the mean of triplicate wells containing tumor cells alone and the background as the mean of triplicate wells containing medium. Non-specific formation of formazan due to the presence of T cells was determined from triplicate wells containing T cells alone in the same number as in the corresponding co-culture wells. Percent of remaining viable tumor cells in the co-culture wells was calculated as follows: viability (%) = [OD (co-culture wells) – OD (T cells alone)]/[OD (tumor cells alone) – OD (medium)] × 100. Cytotoxicity (%) was defined as 100 – viability (%) [1] (OD = optical density).

### Luciferase-based caspase-3/-7 assay

Additional to the real-time live-cell imaging assay, caspase-3/-7 mediated apoptosis of BTICs was quantified using Caspase-Glo® 3/7 Assay Systems (Promega, #G8093). shRNA transduced BTIC13 (bulk or clones) and BTIC129 cells were seeded in a white-opaque 96-well plate (Perkin Elmer, #6005680) and either co-cultured with T cells as described in the XTT-assay or treated with activated supernatant of T cells or 50 ng/ml TRAIL. After 4 h, 100 µl of Caspase-Glo® 3/7 Reagent was added to cells. After 30 min of incubation at room temperature on an orbital shaker (500 rpm), luminescence intensity indicating active caspase-3/-7 levels was measured using a Spark 10 M multimode microplate reader (TECAN). The luminescence intensity measured in T cell only wells was subtracted from co-culture wells to exclude T cell-derived caspase-3/-7 activity.

### Real-time live-cell imaging assay

Caspase-3/-7 mediated apoptosis of BTICs was determined by real-time live-cell imaging using Incucyte® SX5 live cell imager (Sartorius). ShRNA-transduced BTIC13 and BTIC129 cells were either co-cultured with T cells as described for XTT-assay or treated with culture supernatant of T cells or 50 ng/ml TRAIL (PeproTech, #310–04). Afterwards, Incucyte® Caspase-3/7 Green Dye was added to cells (1:1000, Sartorius, #4440). Cells were imaged for the indicated time points at a 10x magnification. Tumor cell apoptosis was quantified with the Incucyte® 2020B software.

### Correlation analysis using The Cancer Genome Atlas and the single-cell RNAseq dataset

GEPIA web server (http://gepia.cancer-pku.cn/) was used to perform correlation analysis between *TSPO* and other indicated genes in The Cancer Genome Atlas (TCGA)-GBM tumor dataset (163 patients) [88]. Pearson`s correlation coefficient (R) and P-value significance were indicated for each correlation.

ScRNA-seq data of a total of 20 adult GB samples profiled by Smart-seq2 were retrieved from the Gene Expression Omnibus (GEO) database through the respective accession number (GSE131928) [65]. The data was filtered and processed using built-in functions in “Seurat” R package as described by the authors [77]. Non-malignant cells were identified by their published markers and further excluded from the analysis while the remaining 4,696 malignant cells were further processed and integrated using the “harmony” R package [44]. 25 PCs were used for clustering at 0.5 resolution and the different subsets of malignant cells were identified upon inspection based on the expression of known marker genes. Pearson correlation coefficient was calculated between *TSPO* and the indicated genes using the data from cells with positive expression values, where cells with zero values of expression were excluded. The “ggpubr” R package was used to display correlation coefficients and P-values on scatter plots [40].

Gene sets (HALLMARK INTERFERON GAMMA RESPONSE, GOBP REGULATION OF CELL DEATH (GO:0010941)) involved in this study were retrieved from the Molecular Signatures Database (MSigDB, http://www.gsea-msigdb.org/gsea/msigdb, v7.5.1) [52].

### Statistical analysis

For statistical analysis, GraphPad Prism software v9.0 (GraphPad Software, La Jolla, CA, USA) was used. Results are reported as mean ± SD (Standard Deviation) as indicated in the figure legends. If not stated otherwise, statistical differences between the control and the test groups were determined by using two-tailed unpaired Student's *t-test*. All Incucyte-based real-time cytotoxicity assay data (Figs. 3e–g; 4e–f; 5 h, l; 6 g; Additional file 1: Supplementary Fig. 1d, 4f) were analyzed using two-tailed paired Student's *t-test*. In all statistical tests, a *P*-value ≤ 0.05 was considered significant with * = *P* ≤ 0.05, ** = *P* ≤ 0.01, *** = *P* ≤ 0.001 and **** = *P* ≤ 0.0001. All experiments with representative images have been repeated at least twice and representative images are shown. For the cumulative data, results from at least three independent experiments were combined.

## Results

### Glioblastoma cells upregulate TSPO expression upon contact with cytotoxic T cells and T cell-derived factors

**Fig. 1:**
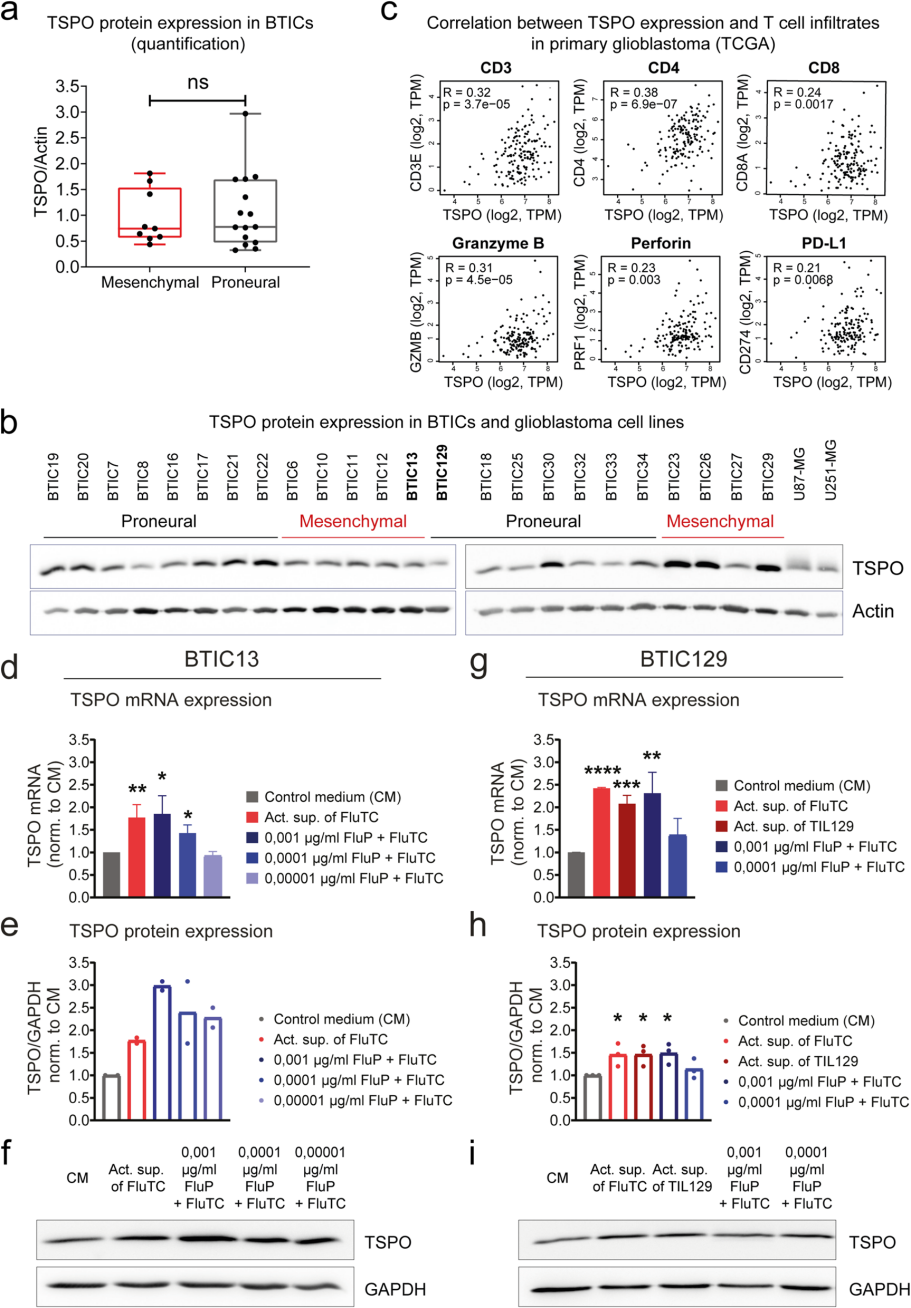
GB cells upregulate TSPO expression upon contact with cytotoxic T cells and T cell-derived factors. **a**, **b** Western blot analysis shows TSPO protein expression in primary BTICs derived from 9 mesenchymal and 15 proneural GB subsets as well as GB cell lines U-87 MG and U-251 MG. **a** TSPO protein quantification of primary BTICs after actin protein normalization. **b** Blots used for the protein quantification indicated in (**a**). **c** Correlation between the expression of *TSPO* and *CD3E*, *CD4*, *CD8A*, *GZMB*, *PRF1*, and *CD274* based on TCGA-GBM dataset. The analysis was performed by GEPIA web server [88]. R indicates Pearson`s correlation coefficient. (TPM: Transcripts per million) **d-i** Induction of TSPO expression in BTICs in response to cytotoxic T cells and T cell-derived factors. The primary cell lines BTIC13 (mesenchymal, **d-f**) and BTIC129 (proneural, **g**-**i**) were treated with supernatant of polyclonally activated FluTC or were co-cultured with FluTC at 1:1 ratio after being pulsed with diluting concentrations of flu-peptide (FluP). BTIC129 cells were additionally treated with supernatant of polyclonally activated TIL129. 20 h following treatment/co-culture, TSPO mRNA and protein expression in the remaining tumor cells were analyzed. **d**, **g** RT-qPCR analysis of *TSPO* mRNA expression in BTICs. Results are presented as fold change compared to the control medium (CM) condition after GAPDH mRNA normalization. **e**, **f**, **h**, **i** Western blot analysis of TSPO protein expression in BTICs. **e**, **h** Protein quantification is presented as fold change compared to the control medium (CM) condition after GAPDH protein normalization. **f**, **i** Representative blot of at least two independent experiments. **d**, **g**, **h** Cumulative data of three and (**e**) two independent experiments. Graphs represent mean ± SD. P-values were calculated using (**a**) Mann–Whitney test (**d, g, h**) two-tailed Student`s t-test (* = *P* < 0.05, ** = *P* < 0.01, *** = *P* < 0.005, **** = *P* < 0.001, ns: non-significant)

In our assays, TSPO protein expression was comparable between a variety of primary mesenchymal and proneural BTICs [61, 62] upon their short-term culture in the absence of immune cells (Fig. 1a-b). On the other hand, TSPO expression in primary GBs correlated with T cell infiltration as analyzed from data of the TCGA-GBM dataset using GEPIA webserver (Fig. 1c) [88]. Correlation analysis revealed a significant positive correlation between *TSPO* and the T cell marker genes *CD3*, *CD4*, *CD8* as well as the cytolytic protein perforin (*PRF1*) and the serine protease Granzyme B (*GZMB*) that are both prevalent in cytotoxic T cells. Furthermore, *TSPO* expression positively correlated with PD-L1 (*CD274*), which is induced in response to the activity of tumor-infiltrating T cells in various tumors [3, 17, 84, 89].

To address whether tumor antigen reactive T cells can trigger TSPO expression, we loaded HLA-A2^+^ BTIC lines BTIC13 (mesenchymal) and BTIC129 (proneural) with an HLA-A2-restricted peptide derived from the Influenza matrix protein M1 (flu-peptide) as a model tumor antigen (influenza matrix protein) and co-cultured them with antigen specific cytotoxic CD8^+^ T cells (FluTC) which we generated from HLA-A2 + healthy donor PBMCs by repetitive antigen stimulation. The generated T cell cultures were highly enriched with flu-specific CD8 + T cells as demonstrated by staining with flu-peptide HLA-A2 pentamer (80%-96%) as well as by functional analysis of their antigen dependent cytokine secretion and lysis of antigen loaded target cells (Additional file 1: Supplementary Fig. 1a–b, e). FluTC-mediated target cell killing was also MHC-I restricted, as blockade of MHC-I on target cells abrogated the lysis of flu-peptide pulsed target cells and cytokine secretion by T cells (Additional File 1: Supplementary Fig. 1c–d, f). After 20 h of co-culture, BTICs upregulated TSPO mRNA and protein where TSPO expression correlated with the applied antigen concentrations, indicating that recognition of the model tumor antigen by T cells was required to induce TSPO (Fig. 1d-i).

Next, we observed comparable upregulation of TSPO expression when we used the culture supernatant of activated FluTC (Fig. 1d-i) or of activated autologous tumor-infiltrating lymphocytes (TILs) isolated from GB 129 (TIL129, Additional File 1: Supplementary Fig. 1g–h) instead of the T cells (Fig. 1g-i). Both supernatants of activated FluTC and TIL129, as well as flu-peptide activated FluTC elevated *TSPO* mRNA levels in BTIC129 up to 2.5-fold. Likewise, TSPO protein levels correlated with mRNA levels in BTICs upon respective treatment (Fig. 1d-i). Notably, *TSPO* mRNA expression did not increase when HLA-A2 negative BTIC131 was co-cultured with FluTC, whereas treatment with the supernatant of activated FluTC or autologous TIL131 elevated *TSPO* levels up to 2.5-fold (Additional File 1: Supplementary Fig. 1i).

### T cell-derived IFNγ and TNFα induce TSPO expression in BTICs

**Fig. 2:**
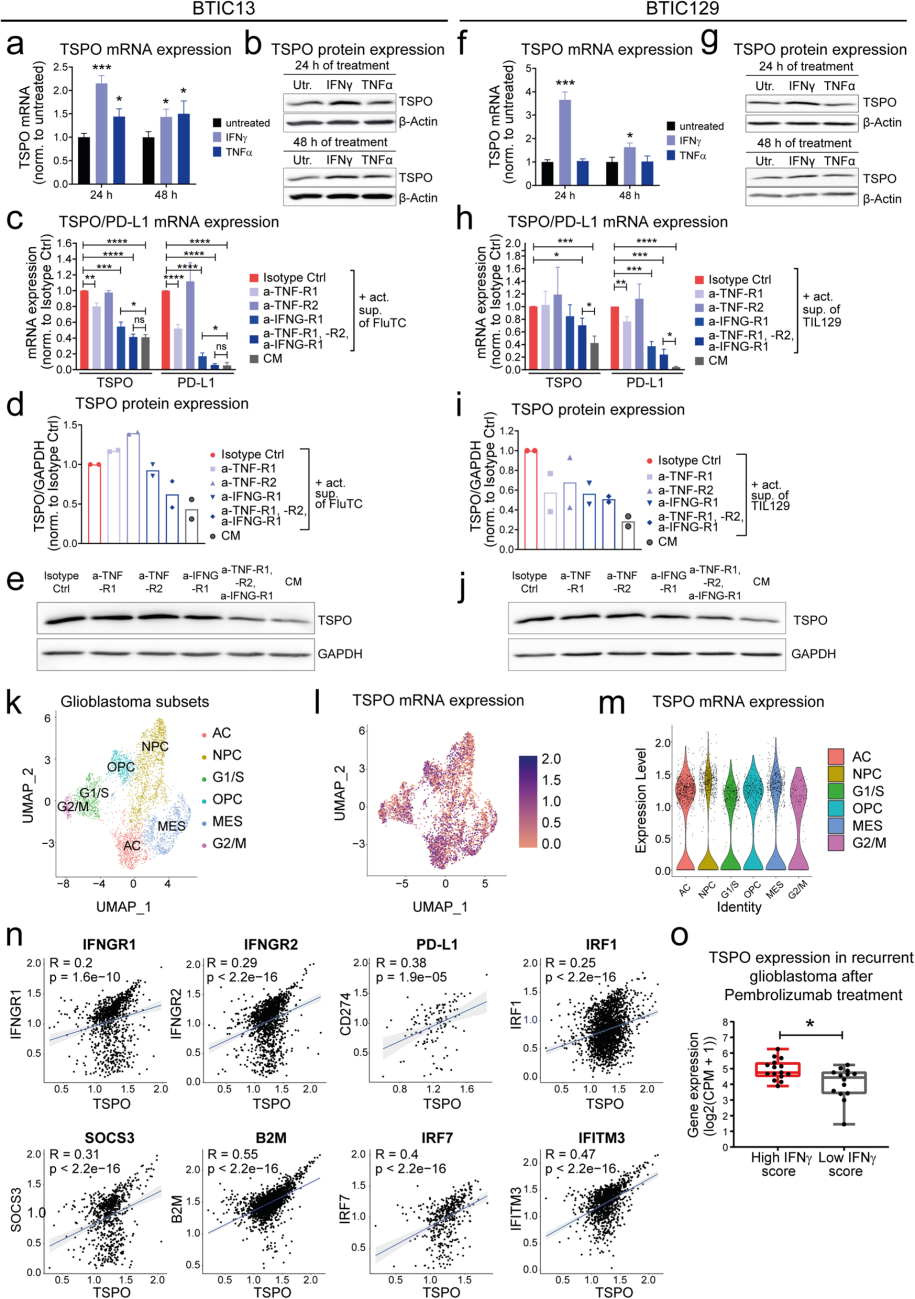
GB cells upregulate TSPO expression upon response to T cell-derived IFNγ and TNFα. **a-j** Induction of TSPO expression in (**a-e**) BTIC13 and in (**f-j**) BTIC129 upon response to TNFα and IFNγ signaling. (**a, b**, **f, g)** BTICs were treated with 50 ng/ml TNFα or IFNγ. 24 and 48 h following treatment TSPO mRNA and protein expression were analyzed. **a, f** RT-qPCR analysis of *TSPO* mRNA expression in BTICs. Results are presented as fold change compared to untreated condition after *RPL13A* mRNA normalization. **b**, **g** Western blot analysis of TSPO protein expression in BTICs. **c**-**e**, **h**-**j** Impact of TNF-R1/2 and IFNG-R1 blockade on TSPO expression in BTICs upon response to activated T cell supernatant. BTICs were pre-incubated with anti-TNF-R1/2 and/or anti-IFNG-R1 blocking antibodies and treated with supernatant of activated (**c-e**) FluTC or (**h-j**) TIL129. Control medium (CM) treatment was used to monitor basal TSPO expression. 24 h following treatment TSPO mRNA and protein expression were analyzed. **c**, **h** RT-qPCR analysis of *TSPO* mRNA expression in BTICs. Results are presented as fold change compared to isotype control after *β-actin* mRNA normalization. **d**, **e**, **i**, **j** Western blot analysis of TSPO protein expression in BTICs. **d**, **i** Protein quantification is presented as fold change compared to isotype control after GAPDH protein normalization. **e**, **j** Representative blot of two independent experiments.** k**-**n** Single-cell RNA-Seq data from 4700 cells sorted from 20 adult GB patients were analyzed for *TSPO* expression and its correlation with IFNγ-induced genes [65]. **k** UMAP (Uniform Manifold Approximation and Projection) plot of all malignant cells. Cells were clustered using the Harmony algorithm and colored on the bases of assigned meta-modules as described in Neftel et al. [65]*.* (AC: Astrocyte-like, NPC: neural-progenitor-like, OPC: oligodendrocyte-progenitor-like, MES: mesenchymal-like, G1/S, G2/M: cycling cells). **l** UMAP plot of *TSPO* expression in malignant cells. **m** Violin plot of *TSPO* expression in all identified clusters. **n** Correlation between the expression of *TSPO* and *IFNGR1*, *IFNGR2* as well as IFNγ-induced genes *CD274*, *IRF1*, *SOCS3*, *B2M*, *IRF7,* and *IFITM3* at single-cell level. R indicates Pearson`s correlation coefficient. **o** Comparison of *TSPO* expression in recurrent GB patients who underwent neoadjuvant or adjuvant Pembrolizumab therapy. RNA-Seq data (GSE121810) from clinical trial NCT02852655 were analyzed to compare TSPO expression in patients with high or low IFNγ scores [18]. (CPM: Counts per million) (**a**, **b**, **d**, **e**, **f**, **g**, **i**, **j**) Representative data of at least two independent experiments are shown. **c, h** Cumulative data of three independent experiments. Values represent the mean ± SD. P-value was calculated using (**a**, **c**, **f**, **h**) two-tailed Student`s t-test and (**o**) Mann–Whitney test (* = *P* < 0.05, ** = *P* < 0.01, *** = *P* < 0.005, **** = *P* < 0.001, ns: non-significant

To delineate soluble T cell-derived factors that can induce TSPO upregulation in BTICs, we studied the TCGA-GBM dataset for genes co-expressed with *TSPO*. *TSPO* expression positively correlated with *IFNGR1*, *IFNGR2, TNFRSF1A,* and *TNFRSF1B* expression in GB (Additional File 1: Supplementary Fig. 2a). Among them, *IFNGR2* ranked among the top 50 genes of the entire transcriptome that positively correlate with *TSPO*. Hypothesizing that signaling through IFNγ and/or TNFα may be related to TSPO expression, we treated BTIC13 and BTIC129 with recombinant IFNγ and TNFα. BTIC13 upregulated TSPO mRNA and protein expression upon IFNγ or TNFα treatment, whereas in BTIC129, TSPO was elevated only in response to IFNγ (Fig. 2a, b, f, g, Additional File 1: Supplementary Fig. 2b, c).

To address whether IFNγ and TNFα were mainly responsible for TSPO upregulation in response to soluble T cell factors of activated T cells, we blocked TNF-R1, TNF-R2, and IFNG-R1 on BTICs prior to treatment with activated T cell supernatants. Besides TSPO, we studied PD-L1 expression as a positive control for effective inhibition of IFNγ- and TNFα-mediated signaling. The blockade of TNF-R1 and IFNG-R1 alone significantly reduced the induction of TSPO and PD-L1 in BTIC13 and the combinatorial blockade of all three receptors completely abrogated TSPO and PD-L1 upregulation, demonstrating an efficient inhibition of cytokine receptor signaling and indicating that TSPO induction by T cells was solely mediated by IFNγ and TNFα (Fig. 2c–e). We performed the same experiment with BTIC129, where we used supernatant of activated autologous TIL129 instead of FluTC (Fig. 2h–j). In line with the previous observation that IFNγ but not TNFα upregulated TSPO in BTIC129, blockade of TNF-R1/2 alone did not affect supernatant-mediated TSPO induction., IFNG-R1 blockade resulted in a slight, although not significant inhibition of TSPO upregulation in BTIC129. However, we observed a significant but still incomplete reduction in TSPO expression after combinatorial receptor blockade. Notably, the blockade of PD-L1 upregulation was also incomplete, suggesting that cytokine receptor interactions were not completely inhibited. In cocultures of BTIC and T cells, we further observed a positive correlation between IFNγ and TNFα levels in the culture medium and TSPO expression in BTICs (Additional File 1: Supplementary Fig. 2d, e).

To corroborate these results, we analyzed a publicly accessible single-cell RNA-Seq dataset including 4,700 GB cells derived from 20 primary GBs (Fig. 2k–n, Additional File 1: Supplementary Fig. 2f-h) [65]. Our analysis demonstrated a broad expression of *TSPO* in all subtypes of GB (Fig. 2k–m). Due to the low number of sorted T cells in this dataset it was not feasible to statistically correlate T cells markers and *TSPO* expression. However, *TSPO* expression in GB cells showed a high correlation with the expression of *IFNGR1*, *IFNGR2, TNFRSF1A,* and *TNFRSF1B* (Fig. 2n, Additional File 1: Supplementary Fig. 2g).

We next performed a correlation analysis between *TSPO* and known IFNγ- and TNFα-induced genes as a potential indicator for IFNγ and TNFα receptor stimulation and found a strong correlation to IFNγ-induced genes (*GBP5, ICAM1, CAMK2D, IRF1, SOCS3, CD44*, and *CCL2*) [74] and to TNF induced genes (*PLAU*, *PLAUR*, *LIF,* and *BIRC3)* (Fig. 2n, Additional File 1: Supplementary Fig. 2f, h) [55, 76]. In accordance, TSPO expression was significantly higher in GBs that developed a marked IFNγ response after neoadjuvant or adjuvant anti-PD-1 therapy compared to GBs with a low IFNγ response after immunotherapy (Fig. 2o) [18].

### TSPO protects glioblastoma cells against T cell-mediated cytotoxicity

**Fig. 3:**
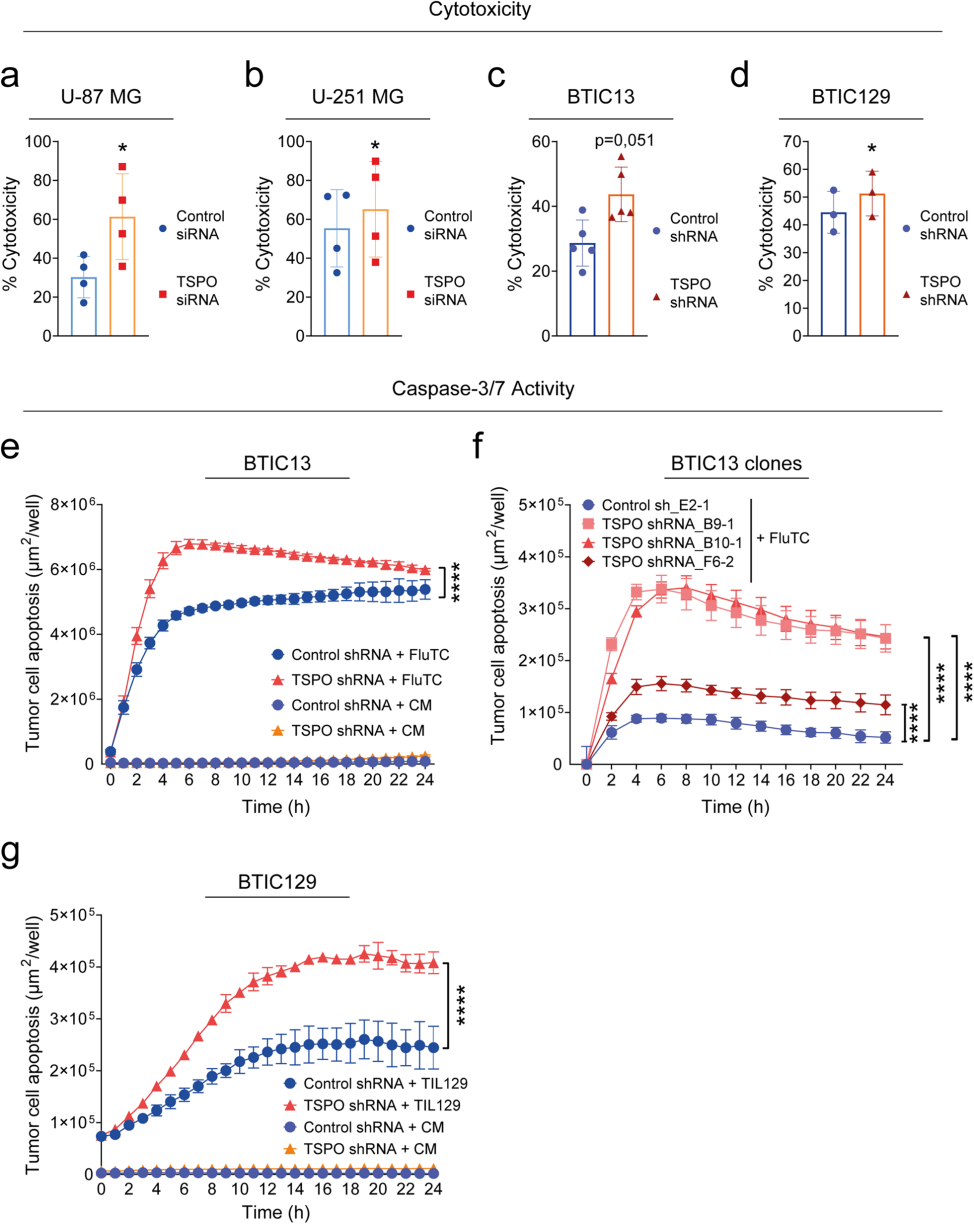
TSPO protects GB cells against T cell-mediated cytotoxicity in both allogeneic and autologous co-culture models. **a**-**d** Tetrazolium salt-based XTT assay to determine the impact of TSPO expression in GB cells on T cell-mediated killing. Human GB cell lines (**a**) U-87 MG and (**b**) U-251 MG were transfected with either control siRNA or a pool of four TSPO-specific non-overlapping siRNAs. After 72 h of transfection, the cells were pulsed with 0.01 μg/ml flu-peptide and cultured with FluTC for 4 h. The primary cell lines BTIC13 (mesenchymal, **c**) and BTIC129 (proneural, **d**) were transduced with either non-targeting control or TSPO-specific shRNA (TSPO-proficient/deficient = TSPO ±). Stable cell lines were seeded on a 96-well plate and co-cultured the next day with (**c**) FluTC after being pulsed with 0.001 μg/ml flu-peptide or (**d**) pre-activated autologous TIL129 for 4 h. Target-cell lysis was determined as described in the Materials and Methods section. **e–g** Real-time cytotoxicity assay (IncuCyte® SX5 System) to analyze T cell-mediated GB cell killing over 24 h. Incucyte® Caspase-3/7 Green Dye was added as an indicator of apoptosis. The graphs show total apoptotic tumor cell area (green object area) per well (µm^2^/well). Co-culture of TSPO ± (**e**) BTIC13, (**f**) BTIC13 clones and (**g**) BTIC129 cells with (**e**, **f**) FluTC and (**g**) pre-activated autologous TIL129. **a-d** Cumulative data of three to five independent experiments, **e–g** Representative data of at least three independent experiments. Values represent the mean ± SD. P-value was calculated using paired two-tailed Student`s t-test (* = *P* < 0.05, ** = *P* < 0.01, *** = *P* < 0.005, **** = *P* < 0.001)

To address the role of TSPO for GB immune resistance, we experimentally silenced TSPO expression in human GB cell lines and primary BTICs and performed cytotoxicity assays using allogeneic and autologous T cells (Fig. 3, Additional File 1: Supplementary Fig. 3). TSPO knockdown caused increased killing of U-87 MG and U-251 MG brain tumor cell lines by FluTC (Fig. 3a, b, Additional File 1: Supplementary Fig. 3a, b). Similarly, TSPO-proficient BTIC13 cells showed a significantly improved resistance against FluTC in comparison to the TSPO-deficient BTIC13 variant (Fig. 3c, Additional File 1: Supplementary Fig. 3c-e). Accordingly, TSPO-deficiency resulted in increased lysis of BTIC129 cells by autologous TILs (Fig. 3d, Additional File 1: Supplementary Fig. 3g, h). These results were confirmed by an alternative method to quantify target cell killing (Fig. 3e–g).

We also generated single-cell TSPO deficient BTIC13 clones and observed increased FluTC-mediated killing in all three TSPO-deficient clones compared to control clone (Fig. 3f, Additional File 1: Supplementary Fig. 3f). Similarly, TSPO-deficient BTIC129 cells showed enhanced apoptosis after coculture with autologous TILs (Fig. 3g). Notably, MHC-I expression was not increased after TSPO silencing, excluding the possibility that improved killing of TSPO-deficient BTICs was related to changes in the immunogenicity of the cells (Additional File 1: Supplementary Fig. 3i).

### TSPO protects glioblastoma cells against cytotoxic molecules secreted by activated T cells

**Fig. 4:**
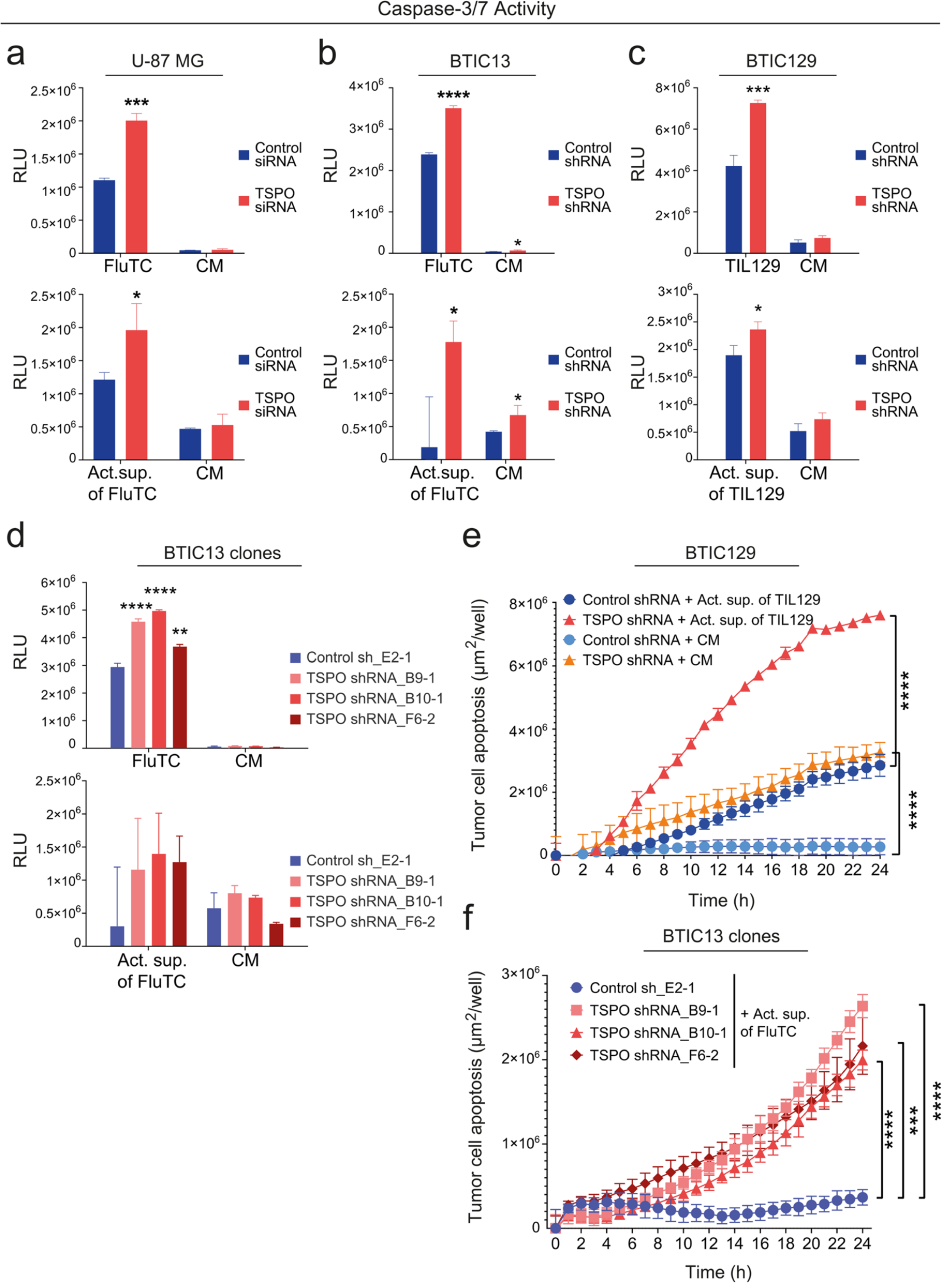
TSPO protects GB cells against cytotoxic molecules secreted by activated T cells. **a-d** Luciferase-based caspase-3/-7 assay to measure caspase-3/-7 activation in TSPO ± (**a**) U-87 MG, (**b**) BTIC13, (**c**) BTIC129 and (**d**) BTIC13 clones after 4 h of co-culture with T cells (upper panel) or treatment with supernatant of polyclonally activated T cells (lower panel). The graphs show background-subtracted raw data in relative luciferase units (RLU). **e, f** Real-time cytotoxicity assay to analyze activated supernatant-induced caspase-3/-7 activation over 24 h in (**e**) BTIC129 and (**f**) BTIC13 clones. The graphs show the total apoptotic tumor cell area (green object area) per well. Co-culture/treatment with (**a, b, d, f**) FluTC or supernatant of activated FluTC, (**c, e**) pre-activated TIL129 or supernatant of activated TIL129. Plain T cell control media (CM) was used to measure the basal level of active caspase-3 /-7. Representative data of at least two independent experiments are shown. Values represent the mean of triplicates ± SD. P-value was calculated using two-tailed Student`s *t-test* (* = *P* < 0.05, ** = *P* < 0.01, *** = *P* < 0.005, **** = *P* < 0.001)

We next investigated whether TSPO can protect GB cells against soluble cytotoxic agents such as death receptor ligands released by cytotoxic T cells. TSPO knockdown caused increased apoptosis as indicated by caspase-3/-7 activation in U-87 MG and BTIC13 cells not only upon co-culture with FluTC, but also after treatment with the supernatant of activated FluTC (Fig. 4a, b, upper and lower panel, respectively). Similarly, TIL129 and their activated supernatant both induced increased apoptosis of TSPO-deficient BTIC129 cells (Fig. 4c, upper and lower panel, respectively). We validated TSPO-mediated protection against cytotoxic molecules in the T cell supernatants by cytotoxicity assays, where TSPO-deficient BTIC13 clones and BTIC129 cells exerted increased apoptosis upon treatment with activated supernatant compared to TSPO-proficient tumor cells (Fig. 4e, f).

### TSPO protects glioblastoma cells from TRAIL-induced apoptosis

In the supernatant of activated FluTC and TIL129, we observed the cytotoxic agents TNFα, IFNγ, FasL, and TRAIL (Additional File 1: Supplementary Fig. 2d, e, 4a, b). An investigation of the respective receptors expressed by BTIC13 and BTIC129 revealed high expression levels of IFNG-R1, Fas and TRAIL-R2 (DR5) and low expression of TNF-R2. TNF-R1 expression was higher on BTIC13, whereas TRAIL-R1 (DR4) was only expressed on BTIC13 (Additional File 1: Supplementary Fig. 4c, d).

**Fig. 5:**
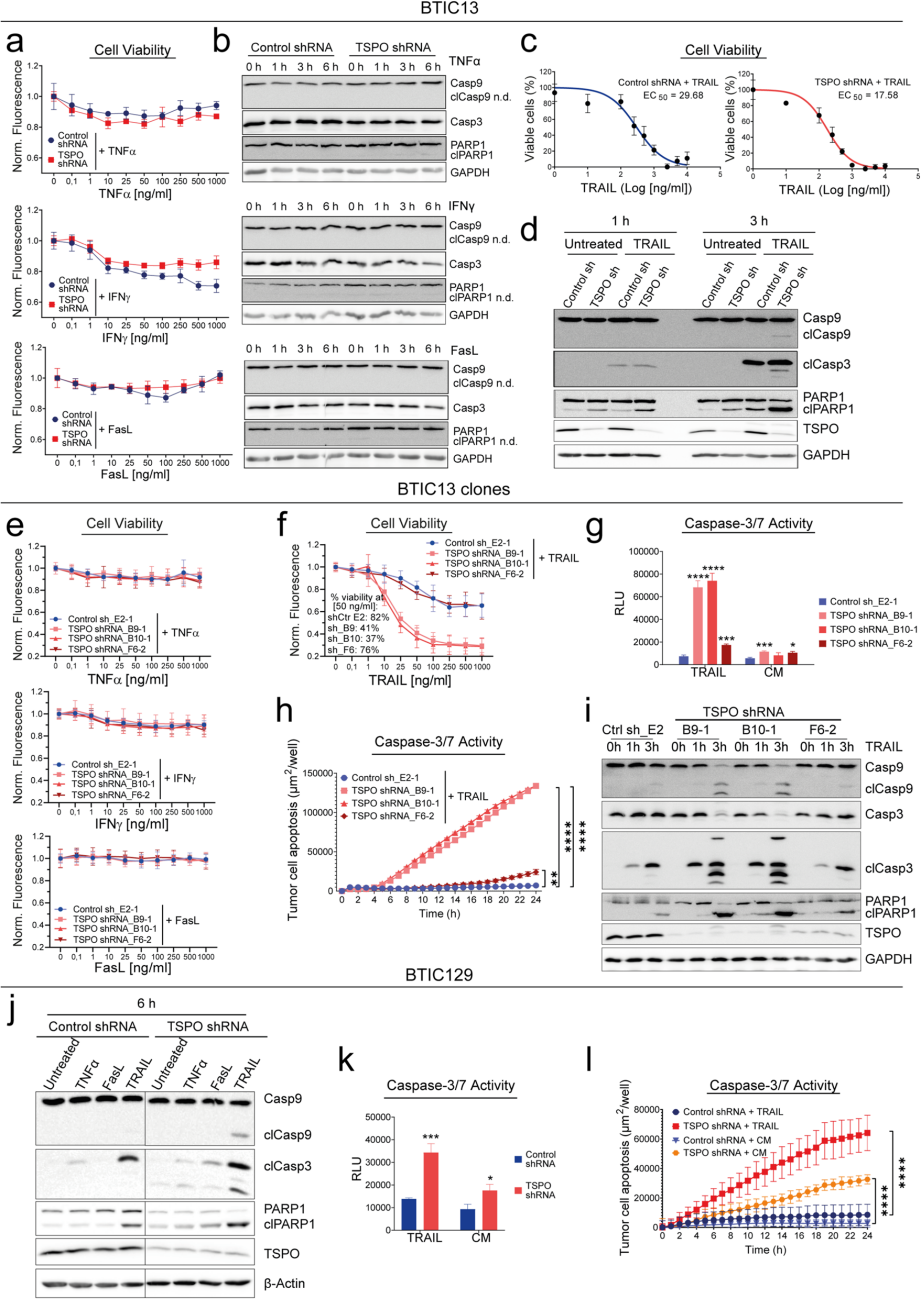
TSPO protects GB cells from TRAIL-induced apoptosis. **a** Resazurin assay to analyze the impact of TNFα, IFNγ, and FasL treatment on the viability of TSPO ± BTIC13 cells. Cells were treated for 48 h and fluorescence was normalized to untreated condition for control or TSPO shRNA-transduced cells. **b** Western blot analysis of total/cleaved caspase-9, total caspase-3, and total/cleaved PARP1 in TSPO ± BTIC13 cells upon 1, 3, and 6 h treatment with 50 ng/ml TNFα, IFNγ, and FasL (cl = cleaved; n.d. = not detected). **c** Resazurin assay to analyze the impact of TRAIL treatment on the viability of TSPO ± BTIC13 cells. Dose–response curves indicate normalized %-cell viability versus log-transformed 50 ng/ml TRAIL concentration. EC_50_ values were determined by nonlinear curve fitting. **d** Western blot analysis of total/cleaved caspase-9/-3 and PARP1 in TSPO ± BTIC13 cells upon 1 and 3 h treatment with TRAIL. **e, f** Resazurin assay to analyze the impact of (**e**) 50 ng/ml TNFα, IFNγ, and FasL and (**f**) TRAIL treatment on the viability of TSPO ± BTIC13 clones. **g** Luciferase-based caspase-3/-7 assay to measure caspase-3/-7 activation in TSPO ± BTIC13 clones after 4 h treatment with 50 ng/ml TRAIL. **h** Real-time cytotoxicity assay to analyze TRAIL-induced caspase-3/-7 activation over 24 h in BTIC13 clones. The graphs show the total apoptotic tumor cell area (green object area) per well. **i, j** Western blot analysis of total/cleaved caspase-9/-3 and PARP1 in TSPO ± (**i**) BTIC13 clones upon 1 and 3 h treatment with 50 ng/ml TRAIL and (**j**) BTIC129 cells upon 6 h treatment with 50 ng/ml TNFα, FasL and TRAIL. **k** Luciferase-based caspase-3/-7 assay to measure caspase-3/-7 activation in TSPO ± BTIC129 cells after 4 h treatment with TRAIL. **l** Real-time cytotoxicity assay to analyze TRAIL-induced caspase-3/-7 activation over 24 h in BTIC129 cells. The graphs show the total apoptotic tumor cell area (green object area) per well. Representative data of at least two independent experiments. **e, f** Cumulative data of three independent experiments. Values represent the mean of triplicates ± SD. P-value was calculated using two-tailed Student`s t-test (* = *P* < 0.05, ** = *P* < 0.01, *** = *P* < 0.005, **** = *P* < 0.001)

Both TSPO-proficient and -deficient BTIC13 cells were resistant to treatment with recombinant human TNFα, IFNγ and FasL, as cell viability was not affected and cleavage of caspase-9/-3 and PARP1 as an indicator of intrinsic/extrinsic apoptosis could not be detected (Fig. 5a, b). However, the sensitivity of BTIC13 against TRAIL-induced apoptosis was increased upon TSPO knockdown, reflected by a reduced EC_50_ value for TRAIL response in TSPO-deficient cells compared to TSPO-proficient cells (Fig. 5c). In accordance, we observed an increased cleavage of caspase-9/-3 and PARP1 in TSPO-deficient cells upon TRAIL treatment, indicating that TSPO was involved in both intrinsic and extrinsic apoptosis pathways (Fig. 5d). We also observed increased caspase-3/-7 activation in TSPO-deficient BTIC13 upon TRAIL treatment (Additional File 1: Supplementary Fig. 4e, f).

In line with these observations, TSPO-deficient BTIC13 clones were resistant against TNFα, IFNγ and FasL while they were sensitive against TRAIL (Fig. 5e–i). A comparison of different TSPO silenced BTIC13 clones suggested that the degree of TSPO expression affects their sensitivity against TRAIL, as clone F6-2, which expressed higher TSPO levels than the other two TSPO-deficient clones, showed a much lower TRAIL sensitivity (Fig. 5i). Similar to BTIC13 cells, BTIC129 cells as well as BTIC111 and BTIC134 cells were selectively protected by TSPO against TRAIL-mediated apoptosis (Fig. 5j–l, Additional File 1: Supplementary Fig. 4g, h).

Since TSPO exerted its tumor protective role through selective protection against TRAIL in vitro, we speculated whether TSPO expression might correlate with TRAIL receptor expression in primary GB. According to both TCGA-GBM and single-cell RNA-Seq datasets, *TSPO* expression in GB cells indeed positively correlates with expression of the TRAIL receptors *TNFRSF10A* (TRAIL-R1) and *TNFRSF10B* (TRAIL-R2) (Additional File 1: Supplementary Fig. 4i, j) [65, 88].

### TSPO regulates the expression of genes associated with resistance against apoptosis

**Fig. 6:**
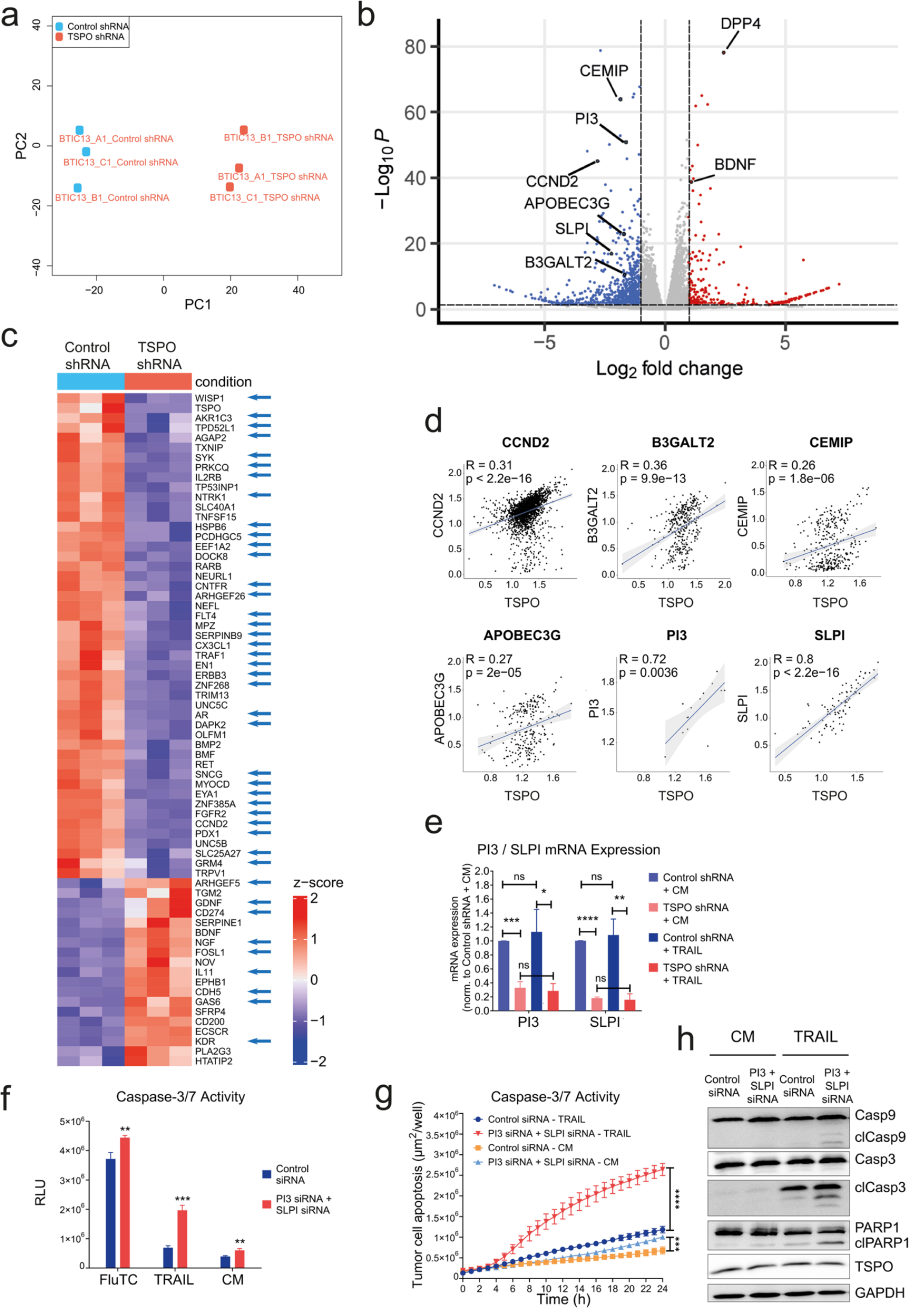
TSPO regulates the expression of genes associated with apoptosis-resistance. **a** Principal component analysis (PCA) of RNA-Seq gene expression profiles from TSPO ± BTIC13. **b** Volcano Plot highlighting differentially expressed genes in TSPO-deficient BTIC13 (fold change ≥ 2, normalized counts per million > 2, false discovery rate (FDR) ≤ 0.05, labelled blue (downregulated) and red (upregulated)). **c** Heatmap of expression changes of DEGs associated with GO term: GOBP_REGULATION_OF_CELL_DEATH (GO:0010941) in TSPO ± BTIC13. Anti-apoptotic genes are indicated with a blue arrow. **d** Correlation between the expression of *TSPO* and *CCDN2*, *B3GALT2*, *CEMIP*, *APOBEC3G*, *PI3*, and *SLPI* at single-cell level [65]. R indicates Pearson`s correlation coefficient. **e** RT-qPCR analysis of *PI3* and *SLPI* mRNA expression in control medium (CM) or TRAIL treated TSPO ± BTIC13. Results are presented as fold change compared to the CM condition of TSPO-proficient cells after *β-actin* mRNA normalization. **f** Luciferase-based caspase-3/-7 assay to measure caspase-3/-7 activation in PI3/SLPI downregulated BTIC13 upon 4 h co-culture with FluTC or treatment with 50 ng/ml TRAIL. **g** Real-time cytotoxicity assay to analyze TRAIL-induced caspase-3/-7 activation over 24 h in BTIC13 cells upon PI3 and SLPI double knockdown. The graphs show the total apoptotic tumor cell area (green object area) per well. **h** Western blot analysis of total/cleaved caspase-9/-3 and PARP1 in BTIC13 transfected with control or PI3-SLPI-specific siRNAs upon treatment with 50 ng/ml TRAIL. **g, h** Values represent the mean of triplicates ± SD. P-value was calculated using two-tailed Student`s t-test (* = *P* < 0.05, ** = *P* < 0.01, *** = *P* < 0.005, **** = *P* < 0.001)

To delineate potential genes associated with TSPO-mediated apoptotic resistance, we conducted next-generation RNA sequencing (RNA-Seq) of TSPO-proficient/deficient BTIC13 and BTIC129. Principal component analysis (PCA) of the transcriptome data showed that sample groups clustered closely based on TSPO expression along the PC1 axis (Fig. 6a, Additional File 1: Supplementary Fig. 5a). Downregulation of TSPO resulted in 728 and 500 differentially expressed genes (DEGs) in BTIC13 and BTIC129, respectively (Fig. 6b, Additional File 1: Supplementary Fig. 5b). We identified 10 common genes, *CCND2, B3GALT2, CEMIP, NDTS4, ABCC9, KCNQ2, CXXC4, ST8SIA5, TXNIP* and *IGFBPL1,* that were downregulated in TSPO-deficient BTIC13 and BTIC129, among which *NDTS4* [34]*, CCND2* [46]*, B3GALT2* [35] and *CEMIP* [50] were previously reported as anti-apoptotic genes (Additional File 2: Supplementary Table 1, Fig. 6b, Additional File 1: Supplementary Fig. 5b). Only four genes, namely *TGM2*, *WNT7B*, *FAM196B* and *EDIL3*, were found to be upregulated both in TSPO-deficient BTIC13 and BTIC129, where *TGM2* [57] was previously reported as a pro-apoptotic gene (Additional File 2: Supplementary Table 1). Additional upregulated pro-apoptotic genes such as *DPP4* [12], *BDNF* [96], *RASSF6* [32, 33] and *ALPK2* [101] as well as downregulated anti-apoptotic genes such as *APOBEC3G* [95]*, PI3* [98] and *SLPI* [56] were revealed in either TSPO-deficient BTIC13 or BTIC129 (Additional File 2: Supplementary Table 1, Fig. 6b, Additional File 1: Supplementary Fig. 5b).

Since it has been shown that TSPO is able to regulate gene expression by mitochondrial retrograde response (MRR), which can result in downregulation of pro-survival NFκB signalling [20, 100], we also determined NF-κB target genes among our anti-apoptotic DEGs. The online tool ChIP-X Enrichment Analysis Version 3 (ChEA3) was used [41]. In TSPO deficient BTIC13, *NTRK1*, *CCND2*, *EEF1A2*, *AGAP2*, *EN1*, *TRAF1*, *CX3CL1*, *IL2RB*, *SERPINB9*, *SYK*, *DOCK8* and *AR* were predicted to be targets of NFKB1/p50, NFKB2/p52, REL, RELA/p65 and/or RELB, whereas in TSPO deficient BTIC129, *CCND2*, *ACKR3*, *NTRK3* and *BMP5* were predicted to be the targets of NF-κB.

A systematic analysis of DEGs associated with GO term “REGULATION OF CELL DEATH” (GO:0010941) (Fig. 6c, Additional Files 1: Supplementary Fig. 5c, 2: Supplementary Table 1) revealed, that in TSPO-deficient BTIC13 the majority of anti-apoptotic genes (33 out of 48) was downregulated (Fig. 6c, Additional File 2: Supplementary Table 1). These analyses suggest that TSPO expression correlates with the acquisition of an anti-apoptotic phenotype in BTIC cells. To explore if such correlation is also found in human GB cells in situ, we analyzed single-cell RNA-Seq dataset from Neftel et al. [65] and observed a positive correlation between *TSPO* expression and all 10 genes that were commonly downregulated in TSPO-deficient BTIC13 and BTIC129. Furthermore, *TSPO* expression correlated with expression of additional anti-apoptotic DEGs (Fig. 6d, Additional File 1: Supplementary Fig. 5d).

Among the DEGs, *PI3* and *SLPI* which encode for protease inhibitors Elafin and Antileukoproteinase were strongly downregulated in TSPO-deficient BTIC13 and showed a high correlation with *TSPO* expression in primary GB in situ. RNA expression analysis validated downregulation of *PI3* and *SLPI* in TSPO-deficient BTIC13 (Fig. 6e). To investigate whether PI3 and SLPI contribute to immune resistance of BTICs, we performed a double knockdown in BTIC13 and analyzed their apoptosis after exposure to FluTC or TRAIL (Fig. 6f–h, Additional File 1: Supplementary Fig. 5e). We observed pronounced caspase-3/-7 activation in PI3/SLPI-deficient BTIC13 upon TRAIL treatment as well as upon co-culture with FluTC (Fig. 6f–g). In addition, cleavage of caspase-9/-3 and PARP1 was increased in PI3/SLPI-deficient cells upon TRAIL treatment, substantiating the tumor-protective role of these two peptidase inhibitors through regulation of both intrinsic and extrinsic apoptosis pathways downstream of TSPO (Fig. 6h).

## Discussion

With the rise of cancer immune therapy, the study of novel immune modulators has become a hotspot of research. In general, GB is resistant to immune therapy, and in particular, resistance to TRAIL-induced apoptosis is a major mechanism by which gliomas escape immune evasion [19, 27, 99]. TSPO has recently gained attention as a diagnostic marker for PET imaging of brain tumors [39] and as a potential therapeutic target due to its upregulation in GB and its correlation with features of malignancy and reduced prognosis [2, 15, 58, 90, 91, 93]. In this study, we therefore addressed the question of a functional role of TSPO in GB cells in the context of inflammation. We focused on the interaction between GB cells and tumor reactive T cells, because cytotoxic T cells have revealed the strongest prognostic impact in many tumors including GB [85] and since their therapeutic use e.g. through immune checkpoint blockade has enabled a fundamental breakthrough in cancer medicine.

We show that human primary BTICs upregulate TSPO upon contact with tumor antigen reactive cytotoxic T cells. TSPO upregulation in the tumor cells required antigen specific activation of T cells interacting with the tumor cells and was initiated through IFNγ and TNFα secreted by activated T cells. Our findings thus suggest that IFNγ and TNFα might mediate TSPO upregulation in GB cells in situ. Analysis of a GB single-cell RNA-Seq dataset revealed a positive correlation between the expression of TSPO and IFNGR/TNFR, as well as IFNγ- and TNFα-induced genes. This might reflect the importance of IFNGR/TNFR signaling on TSPO expression in brain tumor cells. Of note, these deductions are based on a correlative analysis at transcriptome level and thus are hypothetical.

The cellular source of increased TSPO expression in GB is still a matter of debate [67]. In brain tumors, TSPO is expressed by tumor cells, infiltrating immune cells and microglia. Under conditions of inflammation, its expression is more predominant in activated microglia and macrophages [4, 58, 70]. In accordance with the fact that TSPO is expressed in GB and inflammatory cells alike, its expression is highest in GBs of the mesenchymal subtype, which contain the strongest infiltration of T cells, macrophages and microglia [15, 38].

Immune-mediated regulation of TSPO expression in malignant cells has not been addressed so far, yet several groups reported contradicting results in mice and humans: IFNγ and LPS stimulation induced TSPO expression in rodent macrophages and microglia, whereas TSPO expression was strongly downregulated in primary human microglia and monocyte-derived macrophages [69]. Similar results were demonstrated by another group where TSPO expression was strongly downregulated in human macrophages that were differentiated into the pro-inflammatory “M1” phenotype, whereas differentiation into the “M2” phenotype did not alter TSPO expression [64]. Together, these findings suggest that TSPO expression in immune cells and brain tumor cells is regulated through different IFNGR and TNFR downstream signaling pathways.

The characteristics of TSPO regulation are similar to those of the major immune checkpoint molecule PD-L1. Here, we show that similar to PD-L1 [10, 11, 18], TSPO also protects tumor cells against an attack by tumor specific cytotoxic T cells, albeit through a different, complementary mechanism: PD-L1 suppresses the activation and function of a T cell [37], while TSPO reduces the sensitivity of GB towards cytotoxic agents released by the T cell.

We also show that TSPO confers resistance against TRAIL-mediated apoptosis. Resistance to TRAIL-induced apoptosis is recognized as a major mechanism by which gliomas escape immune evasion [19, 27, 99]. Multiple molecular aberrations contribute to TRAlL-resistance of glioma, such as lower expression of caspase-8 [82], overexpression of c-FLIP [71], Bcl-2 [25] and Bcl-xL [63] or loss or structural alterations of TRAIL-R1, TRAIL-R2, Apaf-1, Smac and Bid [49, 97]. We found that TSPO expression protects BTICs from TRAIL-induced apoptosis by interfering with both intrinsic and extrinsic apoptotic cascades and by regulating the expression of genes that contribute to apoptosis-resistance. All tested brain tumor cell lines and BTIC lines displayed increased immune sensitivity upon TSPO silencing. However, it is possible that the occurrence or the degree of TSPO-mediated immune resistance may be affected by the genotypic subtype of GB, which was not addressed in this study.

Until now, studies on the function of TSPO in apoptosis demonstrated contradictory results. Most of these studies used synthetic TSPO ligands, which can act antagonistically or agonistically depending on the model system [2, 72]. Of note, the applied concentrations of TSPO ligands had an impact on the outcome, potentially due to off-target effects [14, 29]. Therefore, we restricted our functional assays to TSPO knockdown tumor cells. Still, other studies by which TSPO was downregulated by genetic manipulation also demonstrated either pro- or anti-apoptotic roles of TSPO depending on the tested cell line [13, 48, 80, 102]. E.g., a pro-apoptotic role of TSPO has been reported in human colorectal cancer cells, C6 rat glioma and U118MG human glioma cells, where TSPO induced reactive oxygen species (ROS) generation and thereby disrupted mitochondrial membrane potentials, subsequently leading to cytochrome c release and formation of a cytosolic apoptosome complex [13, 48, 80, 102]. On the contrary, TSPO deficiency increased the apoptotic rate of GL261 mouse glioma cells as measured by elevated basal caspase-3 activity, a phenotype which we also observed in TSPO-deficient BTICs [26]. Importantly, neither of these studies addressed the function of TSPO in the context of a challenge by cytotoxic T cells. Thus, these reports cannot be easily compared to our findings.

To deeper understand how TSPO confers resistance against TRAIL, we performed transcriptome analyses to investigate whether TSPO regulated the expression of apoptosis-associated genes in BTICs. These studies revealed anti- and pro-apoptotic genes that are down- and upregulated in TSPO-deficient BTICs respectively. Notably, all 10 DEGs found in our studies showed a positive correlation with TSPO expression in malignant GB cells at single cell level [65] and four of them, *NDTS4* [34]*, CCND2* [46]*, B3GALT2* [35]*,* and *CEMIP* [50], were jointly downregulated in both BTIC13 and BTIC129. All of these have reported anti-apoptotic roles in tumors. Additional not jointly regulated, but line-specific DEGs that appeared in our analysis are known to mediate apoptosis resistance in different tumor entities including GB. As an example, *DAPK2* inhibited TRAIL-induced apoptosis in TSPO-deficient BTIC13 and in various tumors [78]. As another example, *ACKR3*, which is only downregulated in TSPO-deficient BTIC129, prevents TMZ-induced apoptosis in glioma [30]. Interestingly, one pro-apoptotic gene, *TXNIP*, was one of the common downregulated DEGs in TSPO-deficient BTIC13 and BTIC129 [104, 106]. Recent studies have shown that TSPO can regulate gene expression via the mitochondrial retrograde response by forming a complex with ACBD3, PKA and AKAP95 [20, 100]. TSPO enables the contact between mitochondria and nucleus and subsequently the transfer of cholesterol into the nucleus. Cholesterol then induces the expression of pro-survival genes by inhibiting NF-κB deacetylation and sustaining its nuclear localization [20]. Further analysis is needed to understand how TSPO might shape the anti-apoptotic gene landscape in BTICs and if e.g. a downregulation of TXNIP compensates for decreased apoptosis-resistance upon TSPO knockdown.

Two peptidase inhibitors, PI3 and SLPI, were among the most strongly downregulated genes in TSPO-deficient BTIC13 and showed a particularly high correlation with TSPO expression in GB cells in situ. Previously, an inhibitory role of PI3 in cisplatin-induced apoptosis has been reported in ovarian cancer cells [98], whereas SLPI inhibited TNFα-induced apoptosis in lymphoma-derived U937 cells [56]; however, a role for apoptosis regulation in brain tumors has not been addressed before. Our data suggest, for the first time, that PI3 and SLPI deficiency may sensitize BTICs towards T-cell and TRAIL-mediated apoptosis, suggesting that TSPO mediates its anti-apoptotic role at least partially by driving the expression of PI3 and SLPI in GB.

Taken together, we here propose that TSPO expression in GB functions as an adaptive immune resistance mechanism, which is co-regulated with PD-L1 expression through the activity of tumor reactive T cells and accessory inflammatory cells and regulates the sensitivity of GB cells towards TRAIL-mediated apoptosis conferred by cytotoxic T cells. Our data indicate that targeting TSPO may be able to sensitize GB to immune cell-mediated cytotoxicity by circumventing tumor intrinsic TRAIL-resistance. Since synthetic TSPO ligands regulating TSPO activity have been developed, pharmacologic TSPO inhibition could be envisaged as a combination immunotherapeutic strategy for the treatment of GB.

## Supplementary Information

**Additional file 1**. Supplementary Figures 1–5.

**Additional file 2: Supplementary Table 1**. TSPO-regulated DEGs with reported role in apoptosis / cell survival.

## Data Availability

All relevant data are included in this article or in the Suppl. information documents. RNA sequencing data have been deposited in the Gene Expression Omnibus (GEO) public functional genomic data repository under accession code GSE210654 (https://www.ncbi.nlm.nih.gov/geo/query/acc.cgi?acc=GSE210654). Data are available upon personal request to the corresponding authors.
