# Supplementary material for: TSPO acts as an immune resistance gene involved in the T cell mediated immune control of glioblastoma
Source: Acta Neuropathol Commun. 2023 May 8;11:75. doi: 10.1186/s40478-023-01550-9 (PMC10165826; doi:10.1186/s40478-023-01550-9)
Supplement: Supplementary file 1 — Additional file 1. Supplementary Figures 1–5. [file 40478_2023_1550_MOESM1_ESM.docx]

# Supplementary Material


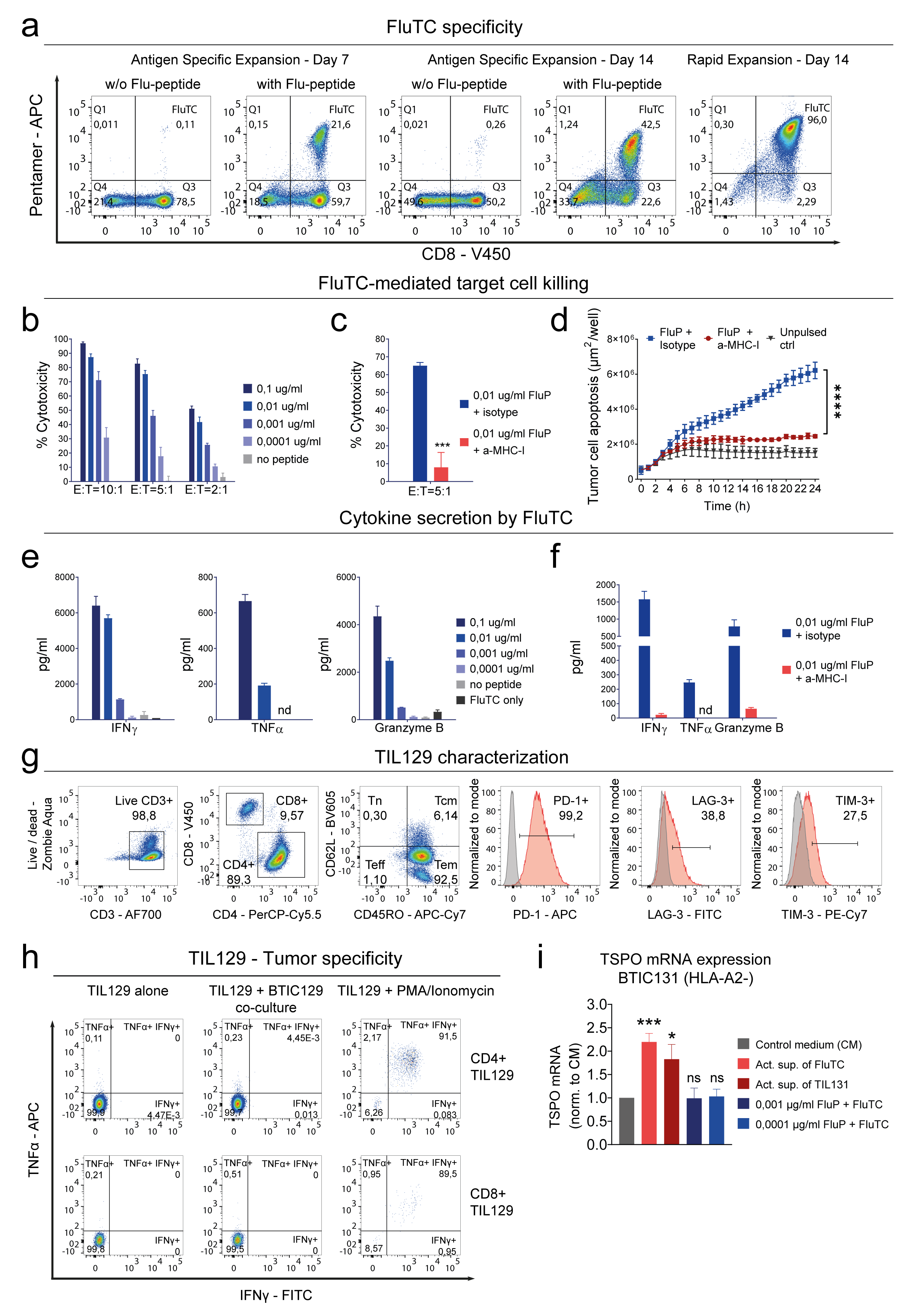


**Suppl. Fig. 1** Glioblastoma-T cell co-culture model. **a** FACS analysis of antigen specificity of FluTC generated from HLA-A2^+^ healthy donors by repetitive expansions. All samples were gated on lymphocytes, single cells, and live cells. CD8 and Flu-Pentamer stainings were performed on days 7 and 14 of antigen-specific expansion (ASE) and day 14 of rapid expansion protocol (REP). During ASE, some CD8^+^ T cells were expanded in the presence of unpulsed feeder cells (w/o flu-peptide) as a negative control. **b-d** Antigen-specific killing of target cells by FluTC. (**b)** XTT assay to determine the cytotoxicity window of FluTC. Human oligodendrocyte cell line MO3.13-A2 cells were pulsed with diluting concentrations of flu-peptide and co-cultured with FluTC at different effector to target (E:T) ratios. After 4 h of co-culture, the percentage of specific target-cell lysis was determined as described in Materials and Methods (**c**) XTT assay to determine MHC-I - TCR engagement-restricted killing of FluTC. Pulsed MO3.13-A2 cells were incubated with MHC-I blocking antibody or isotype control prior to co-culture and cytotoxicity was determined as described in (**b**). (**d)** Real-time cytotoxicity assay (IncuCyte®) to analyze MHC-I - TCR engagement-restricted killing of MO3.13-A2 cells by FluTC. Pulsed MO3.13-A2 cells were co-cultured with FluTC in the presence of an MHC-I blocking antibody or isotype control for 24 h. The graph depicts the total apoptotic tumor cell area as determined by YOYO®-1 dye incorporation. **e, f** ELISA for detecting IFNγ, TNFα, and Granzyme B secretion by FluTC upon 24 h co-culture with MO3.13-A2 cells (**e**) pulsed with titrating concentrations of flu-peptide and (**f**) in the presence of an MHC-I blocking antibody or isotype control. **g** Phenotypical characterization of TIL129 after REP. Expression of T cell markers CD4 and CD8, effector and memory markers CD45RO and CD62L, and activation/exhaustion markers PD-1, LAG-3, and TIM-3 were gated on live CD3^+^ TIL129. Tn: Naïve T cells; Tcm: central memory, Tem: effector memory; Teff: terminal effector cells. Grey histograms represent isotype control. **h** IFNγ and TNFα cytokine secretion assay to determine autologous tumor-reactive TIL percentage. TIL129 were co-cultured with BTIC129 for 12 h prior to staining. Unstimulated TIL129 and PMA/Ionomycin stimulated TIL129 were used as negative and positive controls respectively. Gating on CD4^+^ (upper panel) and CD8^+^ (lower panel) TIL129. **i** RT-qPCR analysis of TSPO mRNA expression in HLA-A2^-^ BTIC131 upon treatment with supernatant of activated FluTC/TIL131 or co-culture with FluTC for 24 h. Results were presented as fold change compared to the control medium (CM) condition after GAPDH mRNA normalization. **a-h** Representative data of at least two independent experiments. **i** Cumulative data of three independent experiments. Graphs represent mean ± SD. P-values were calculated using two-tailed Student`s t-test (* = *P* < 0.05, ** = *P* < 0.01, *** = *P* < 0.005, **** = *P* < 0.001, ns: non-significant, nd = not detected)


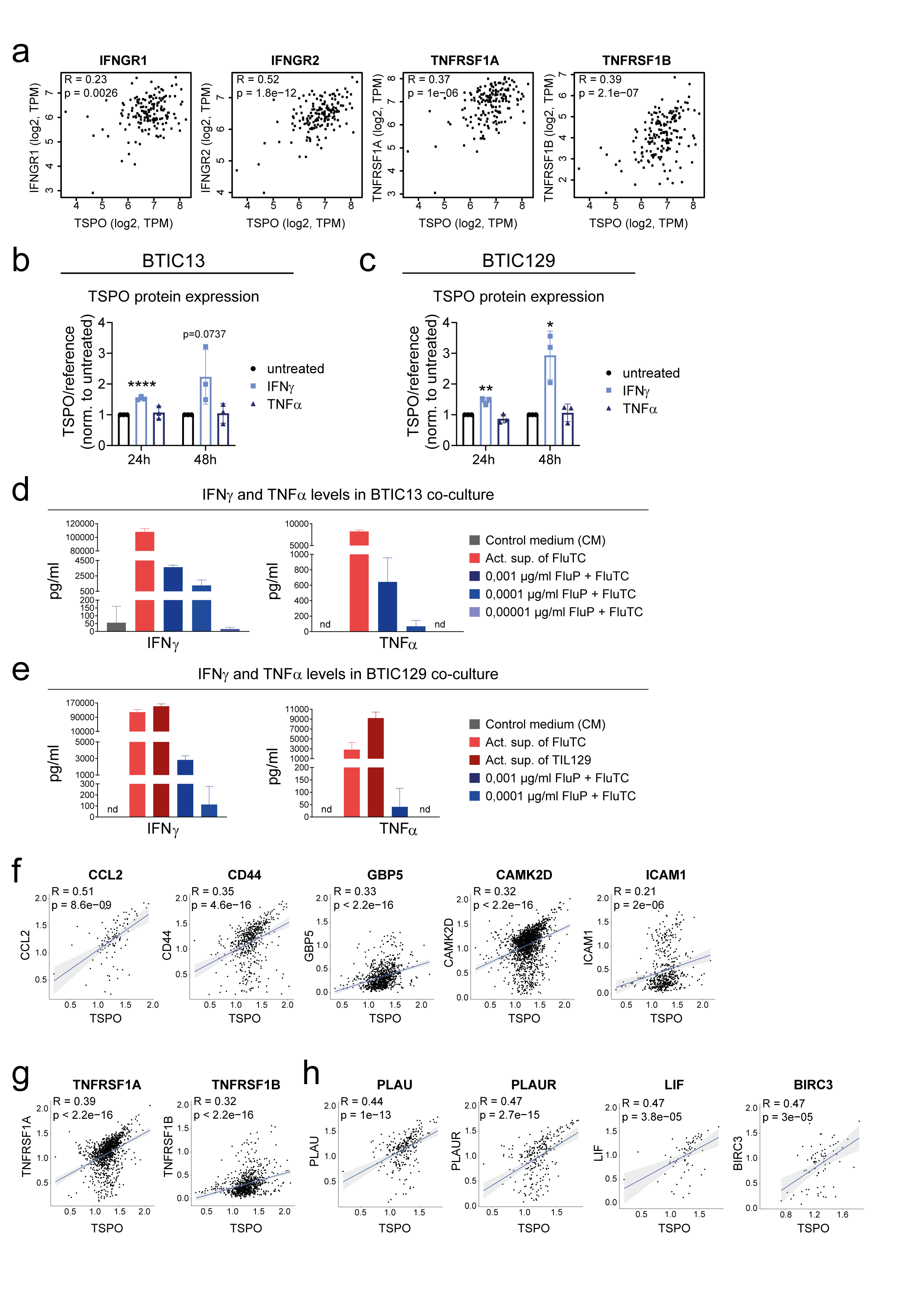


**Suppl. Fig. 2** Additional correlation analysis between *TSPO* and genes induced by TNFα and IFNγ. **a** Correlation between *TSPO* and *IFNGR1*, *IFNGR2*, *TNFRSF1A,* and *TNFRSF1B* expression in glioblastoma performed by GEPIA web server [2]. R indicates Pearson`s correlation coefficient. (TPM: Transcripts per million) **b, c** Western blot analysis of TSPO protein expression in (**b**) BTIC13 and (**c**) BTIC129 upon response to TNFα and IFNγ. Protein quantification is presented as fold change compared to isotype control after reference protein normalization. **d, e** ELISA to determine IFNγ and TNFα levels in the treatments/co-cultures represented in Figures 1D and 1G. **f, g** Correlation between the expression of *TSPO* and (**f**) further IFNγ-induced genes *CCL2, CD44, GBP5, CAMK2D, and ICAM1,* (**g**) *TNFRSF1A* and *TNFRSF1B*, (**h**) TNFα-induced genes *PLAU, PLAUR, LIF* and *BIRC3* at single-cell level [1]. R indicates Pearson`s correlation coefficient. **b-e** Cumulative data of three independent experiments. Values represent the mean ± SD. P-value was calculated using two-tailed Student`s t-test (* = *P* < 0.05, ** = *P* < 0.01, *** = *P* < 0.005, **** = *P* < 0.001, nd = not detected)


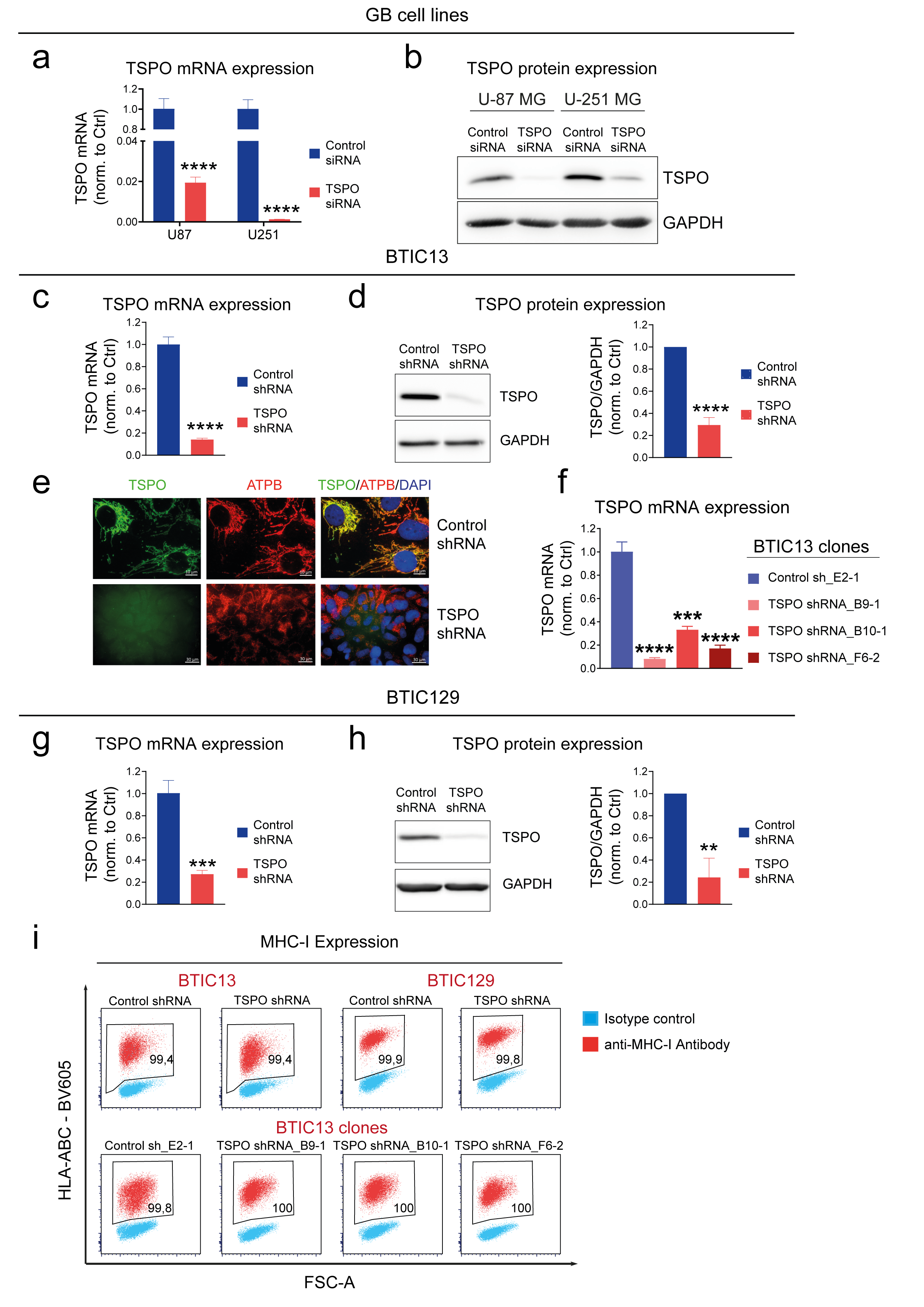


**Suppl. Fig. 3** Knockdown efficiency of TSPO-specific siRNAs/shRNA in glioblastoma cells. **a** RT-qPCR analysis of TSPO knockdown in U-87 MG and U-251 MG cell lines at mRNA level. Cells were reverse transfected with either non-targeting control siRNA or a pool of 4 TSPO-specific non-overlapping siRNAs for 3 days. Results are presented as fold change compared to control siRNA transfected cells after β-actin mRNA normalization. **b** Western blot analysis of TSPO knockdown in U-87 MG and U-251 MG cells at protein level following siRNA transfection as described in (**a**). **c-h** Knockdown efficiency of TSPO shRNA in BTICs. (**c, f, g**) RT-qPCR analysis of TSPO knockdown in (**c**) BTIC13, (**f**) BTIC13 clones, and (**g**) BTIC129 cells that were transduced with either non-targeting control shRNA or TSPO-specific shRNA. Results are presented as fold change compared to control shRNA-transduced cells after β-actin mRNA normalization. **d, h** Western blot analysis of TSPO knockdown in BTICs. Left panel: representative blot, right panel: protein quantification of three independent blots represented as fold change compared to control shRNA-transduced cells after GAPDH normalization. **e** Fluorescence microscopy to determine the cellular localization of TSPO in control or TSPO shRNA-transduced BTIC13 cells. ATPB and DAPI stains were performed for mitochondrial and nuclear localization respectively. **i** FACS analysis for MHC-I expression on TSPO +/- BTIC13, BTIC13 clones, and BTIC129. Blue and red dot plots indicate isotype control and anti-MHC-I antibody staining, respectively. The percentage of MHC-I expressing cells among live cells are indicated in the gate. Representative data of at least two independent experiments are shown. Values represent mean ± SD. P-values were calculated using two-tailed Student`s t-test (* = *P* < 0.05, ** = *P* < 0.01, *** = *P* < 0.005, **** = *P* < 0.001). ATPB: F(1)F(0) ATP synthase


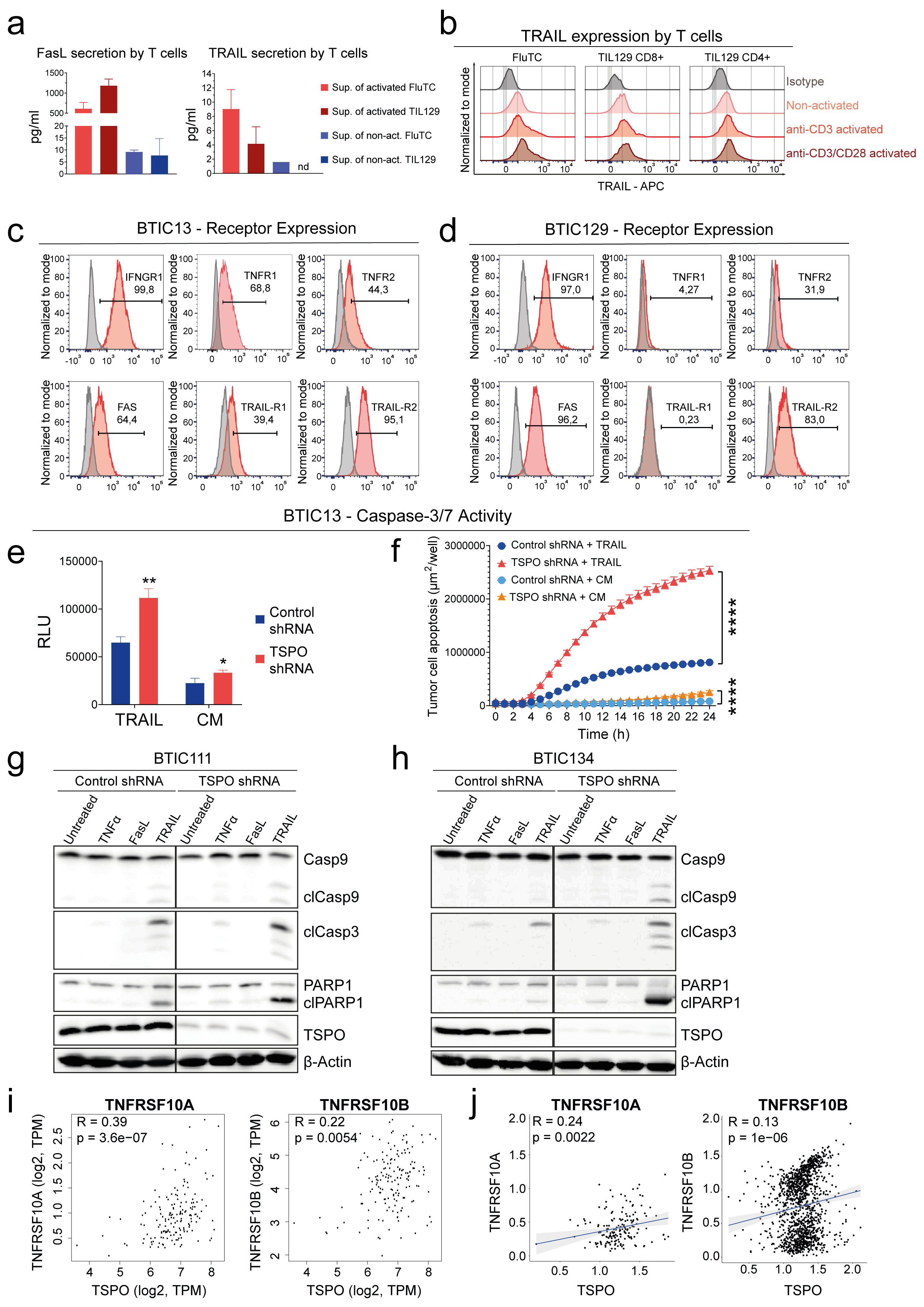


**Suppl. Fig. 4** Further analysis of the impact of TSPO on TRAIL-resistance of glioblastoma cells. **a** Multiplex cytokine assay (left) and ELISA (right) to determine FasL and TRAIL secretion by anti-CD3/CD28 activated and non-activated FluTC and TIL129. **b** FACS analysis of the surface expression of TRAIL on non-activated, anti-CD3, and anti-CD3/CD28 activated FluTC and CD4^+^/CD8^+^ TIL129. **c, d** FACS analysis of the surface expression of IFNG-R1, TNF-R1, TNF-R2, Fas, TRAIL-R1, and TRAIL-R2 on (**c**) BTIC13 and (**d**) BTIC129 cells. Grey histograms represent the isotype control. **e** Luciferase-based caspase-3/-7 assay to measure caspase-3/-7 activation in TSPO +/- BTIC13 cells after 4 h treatment with TRAIL. **f** Real-time cytotoxicity assay to analyze TRAIL-induced caspase-3/-7 activation over 24 h in BTIC13 cells. The graphs show the total apoptotic tumor cell area (green object area) per well. **g, h** Western blot analysis of total/cleaved caspase-9/-3 and PARP1 in TSPO +/- (**g**) BTIC111 and (**h**) BTIC134 cells upon treatment with TNFα, FasL and TRAIL. **i** Correlation between the expression of *TSPO* and TRAIL receptors *TNFRSF10A* and *TNFRSF10B* in glioblastoma. The analysis was performed by GEPIA web server [2]. R indicates Pearson`s correlation coefficient. **j** Correlation between the expression of *TSPO* and *TNFRSF10A* and *TNFRSF10B* at single-cell level [1]. R indicates Pearson`s correlation coefficient. Values represent the mean of triplicates ± SD. P-value was calculated using two-tailed Student`s t-test (* = *P* < 0.05, ** = *P* < 0.01, *** = *P* < 0.005, **** = *P* < 0.001, nd = not detected)


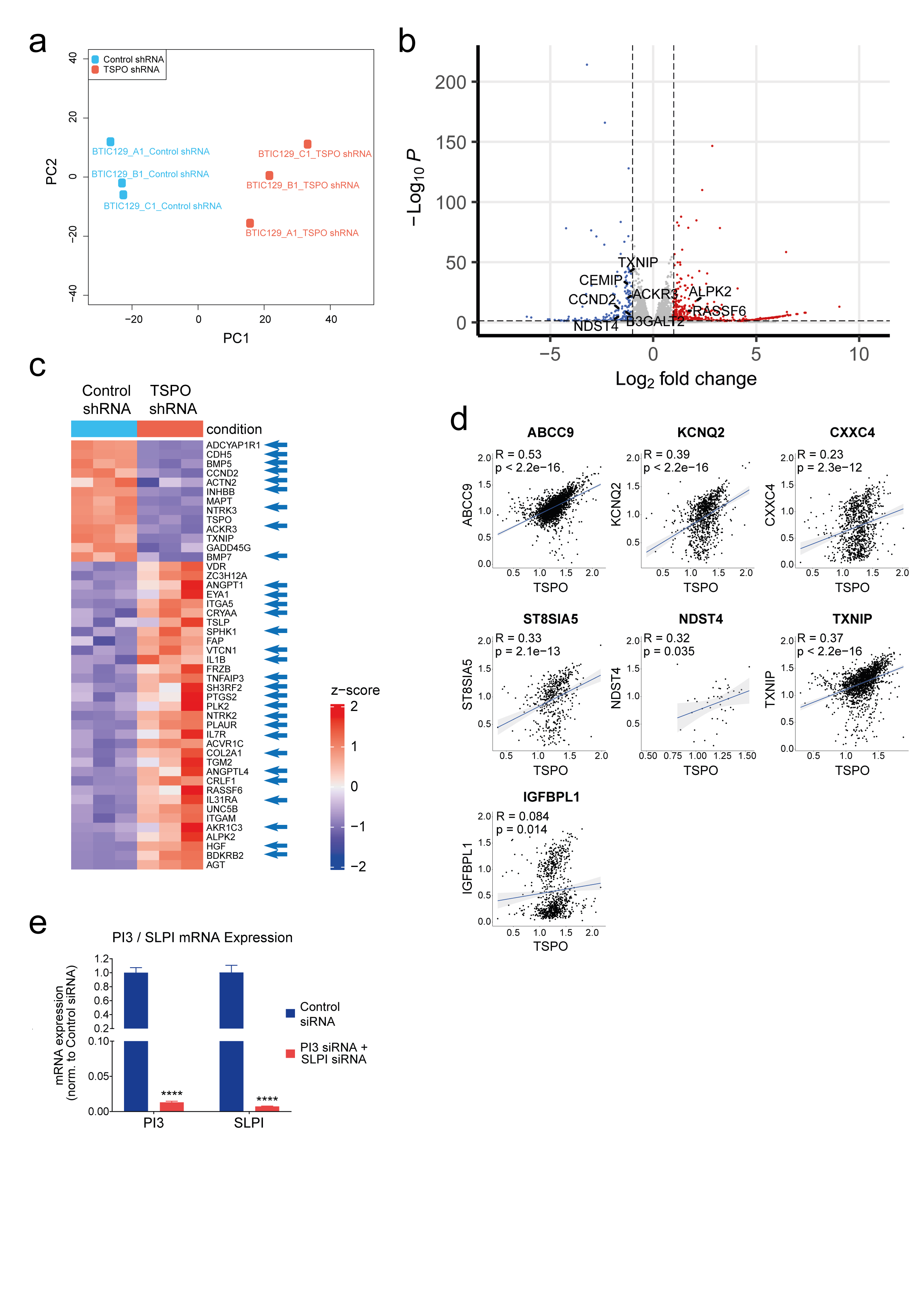


**Suppl. Fig. 5** TSPO regulates the expression of genes associated with apoptosis-resistance. **a** Principal component analysis (PCA) of RNA-Seq gene expression profiles from TSPO +/- BTIC129. **b** Volcano Plot highlighting differentially expressed genes in TSPO deficient BTIC129 (fold change ≥ 2, normalized counts per million > 2, false discovery rate (FDR) ≤ 0.05, labelled blue (downregulated) and red (upregulated)). **c** Heatmap of expression changes of DEGs associated with GO term: GOBP_REGULATION_OF_CELL_DEATH (GO:0010941) in TSPO +/- BTIC129. Anti-apoptotic genes are indicated with blue arrows. **d** Correlation between the expression of *TSPO* and *ABCC9, KCNQ2, CXXC4, ST8SIA5, NDST4, TXNIP* and *IGFBPL1* at single-cell level [1]. R indicates Pearson`s correlation coefficient. **e** Knockdown efficiency of PI3 and SLPI specific siRNAs in BTIC13 as determined by RT-qPCR. Values represent the mean of triplicates ± SD. P-value was calculated using two-tailed Student`s t-test (* = *P* < 0.05, ** = *P* < 0.01, *** = *P* < 0.005, **** = *P* < 0.001)

References

1. Neftel C, Laffy J, Filbin MG et al. (2019) An Integrative Model of Cellular States, Plasticity, and Genetics for Glioblastoma. Cell 178:835-849 e21. doi: 10.1016/j.cell.2019.06.024

2. Tang Z, Li C, Kang B et al. (2017) GEPIA: a web server for cancer and normal gene expression profiling and interactive analyses. Nucleic Acids Res 45:W98-W102. doi: 10.1093/nar/gkx247
