# Supplementary material for: TSPO acts as an immune resistance gene involved in the T cell mediated immune control of glioblastoma
Source: Acta Neuropathol Commun. 2023 May 8;11:75. doi: 10.1186/s40478-023-01550-9 (PMC10165826; doi:10.1186/s40478-023-01550-9)
Supplement: Supplementary file 2 — Additional file 2: Supplementary Table 1. TSPO-regulated DEGs with reported role in apoptosis / cell survival. [file 40478_2023_1550_MOESM2_ESM.docx]

**Supplementary Table 1**

**TSPO-regulated DEGs with reported role in apoptosis / cell survival**

| **Gene** | **regulation in TSPO deficient cells** | | **Reference** |
| --- | --- | --- | --- |
| **Anti-apoptotic DEGs in TSPO-deficient BTIC13 (associated to GO term: regulation of cell death)** | | | |
| AGAP2 | downregulated in BTIC13 | | 1 |
| AKR1C3 | downregulated in BTIC13 (upregulated in BTIC129) | | 2 |
| AR | downregulated in BTIC13 | | 3 |
| ARHGEF26 | downregulated in BTIC13 | | 4 |
| ARHGEF5 | upregulated in BTIC13 | | 5 |
| CD274 | upregulated in BTIC13 | | 6 |
| CDH5 | upregulated in BTIC13 (downregulated in BTIC129) | | 7 |
| CNTFR | downregulated in BTIC13 | | 8 |
| CX3CL1 | downregulated in BTIC13 | | 9 |
| DAPK2 | downregulated in BTIC13 | | 10,11 |
| DOCK8 | downregulated in BTIC13 | | 12 |
| EEF1A2 | downregulated in BTIC13 | | 13 |
| EN1 | downregulated in BTIC13 | | 14 |
| ERBB3 | downregulated in BTIC13 | | 15,16 |
| EYA1 | downregulated in BTIC13 (upregulated in BTIC129) | | 17 |
| FGFR2 | downregulated in BTIC13 | | 18 |
| FLT4 | downregulated in BTIC13 | | 19 |
| FOSL1 | upregulated in BTIC13 | | 20 |
| GAS6 | upregulated in BTIC13 | | 21 |
| GDNF | upregulated in BTIC13 | | 22 |
| GRM4 | downregulated in BTIC13 | | 23 |
| HSPB6 | downregulated in BTIC13 | | 24 |
| IL11 | upregulated in BTIC13 | | 25 |
| IL2RB | downregulated in BTIC13 | | 26 |
| KDR | upregulated in BTIC13 | | 27 |
| MPZ | downregulated in BTIC13 | | 28 |
| MYOCD | downregulated in BTIC13 | | 29 |
| NGF | upregulated in BTIC13 | | 30 |
| NTRK1 | downregulated in BTIC13 | | 31 |
| PCDHGC5 | downregulated in BTIC13 | | 32 |
| PDX1 | downregulated in BTIC13 | | 33 |
| PRKCQ | downregulated in BTIC13 | | 34 |
| SERPINB9 | downregulated in BTIC13 | | 35 |
| SLC25A27 | downregulated in BTIC13 | | 36 |
| SNCG | downregulated in BTIC13 | | 37 |
| SYK | downregulated in BTIC13 | | 38 |
| TPD52L1 | downregulated in BTIC13 | | 39 |
| TRAF1 | downregulated in BTIC13 | | 40 |
| UNC5B | downregulated in BTIC13 (upregulated in BTIC129) | | 41 |
| WISP1 | downregulated in BTIC13 | | 42 |
| ZNF268 | downregulated in BTIC13 | | 43 |
| ZNF385A | downregulated in BTIC13 | | 44 |
| **Selected anti-apoptotic DEGs in TSPO-deficient BTIC13 based on literature search** | | | |
| ABCC6 | downregulated in BTIC13 | | 45 |
| APOBEC3G | downregulated in BTIC13 | | 46 |
| ARHGAP9 | downregulated in BTIC13 | | 47 |
| DMBX1 | downregulated in BTIC13 | | 48 |
| PI3 | downregulated in BTIC13 | | 49 |
| SLPI | downregulated in BTIC13 | | 50 |
| TNFSF13 | downregulated in BTIC13 | | 51 |
| **Anti-apoptotic DEGs in TSPO-deficient BTIC129 (associated to GO term: regulation of cell death)** | | | |
| ACKR3 | downregulated in BTIC129 | | 52 |
| ACTN2 | downregulated in BTIC129 | | 53 |
| ADCYAP1R1 | downregulated in BTIC129 | | 54 |
| AKR1C3 | upregulated in BTIC129 (downregulated in BTIC13) | | 2 |
| ANGPT1 | upregulated in BTIC129 | | 55 |
| ANGPTL4 | upregulated in BTIC129 | | 56 |
| BDKRB2 | upregulated in BTIC129 | | 57 |
| BMP5 | downregulated in BTIC129 | | 58 |
| BMP7 | downregulated in BTIC129 | | 59 |
| CDH5 | downregulated in BTIC129 (upregulated in BTIC13) | | 7 |
| COL2A1 | upregulated in BTIC129 | | 60 |
| CRLF1 | upregulated in BTIC129 | | 61 |
| CRYAA | upregulated in BTIC129 | | 62 |
| EYA1 | upregulated in BTIC129 (downregulated in BTIC13) | | 17 |
| HGF | upregulated in BTIC129 | | 63 |
| IL1B | upregulated in BTIC129 | | 64 |
| IL31RA | upregulated in BTIC129 | | 65 |
| IL7R | upregulated in BTIC129 | | 66 |
| INHBB | downregulated in BTIC129 | | 67 |
| ITGA5 | upregulated in BTIC129 | | 68 |
| NTRK2 | upregulated in BTIC129 | | 69 |
| NTRK3 | downregulated in BTIC129 | | 70 |
| PLAUR | upregulated in BTIC129 | | 71 |
| PLK2 | upregulated in BTIC129 | | 72 |
| PTGS2 | upregulated in BTIC129 | | 73 |
| SH3RF2 | upregulated in BTIC129 | | 74 |
| SPHK1 | upregulated in BTIC129 | | 75 |
| TNFAIP3 | upregulated in BTIC129 | | 76 |
| VTCN1 | upregulated in BTIC129 | | 77 |
| **Common TSPO-regulated DEGs in BTIC13 and BTIC129** | | | |
| **Gene** | **regulation in TSPO deficient cells** | **reported role in apoptosis** | **Reference** |
| ABCC9 | downregulated in BTIC13 & BTIC129 | n.a. | - |
| B3GALT2 | downregulated in BTIC13 & BTIC129 | Anti-apoptotic | 78 |
| CCND2^a^ | downregulated in BTIC13 & BTIC129 | Anti-apoptotic | 79 |
| CEMIP | downregulated in BTIC13 & BTIC129 | Anti-apoptotic | 80,81 |
| CXXC4 | downregulated in BTIC13 & BTIC129 | n.a. | - |
| IGFBPL1 | downregulated in BTIC13 & BTIC129 | n.a. | - |
| KCNQ2 | downregulated in BTIC13 & BTIC129 | n.a. | - |
| NDST4 | downregulated in BTIC13 & BTIC129 | Anti-apoptotic | 82 |
| ST8SIA5 | downregulated in BTIC13 & BTIC129 | n.a. | - |
| TXNIP^a^ | downregulated in BTIC13 & BTIC129 | Pro-apoptotic | 83,84 |
| EDIL3 | upregulated in BTIC13 & BTIC129 | Anti-apoptotic | 85 |
| FAM196B | upregulated in BTIC13 & BTIC129 | Anti-apoptotic | 86 |
| TGM2^a^ | upregulated in BTIC13 & BTIC129 | Pro-apoptotic | 87 |
| WNT7B | upregulated in BTIC13 & BTIC129 | Anti-apoptotic | 88 |

^a^Associated to GO term: regulation of cell death.

**References**

1. Ahn JY, Hu Y, Kroll TG, Allard P, Ye K. PIKE-A is amplified in human cancers and prevents apoptosis by up-regulating Akt. *Proc Natl Acad Sci U S A*. May 4 2004;101(18):6993-8. doi:10.1073/pnas.0400921101

2. Zhou Q, Tian W, Jiang Z*, et al*. A Positive Feedback Loop of AKR1C3-Mediated Activation of NF-kappaB and STAT3 Facilitates Proliferation and Metastasis in Hepatocellular Carcinoma. *Cancer Res*. Mar 1 2021;81(5):1361-1374. doi:10.1158/0008-5472.CAN-20-2480

3. Lamb LE, Zarif JC, Miranti CK. The androgen receptor induces integrin alpha6beta1 to promote prostate tumor cell survival via NF-kappaB and Bcl-xL Independently of PI3K signaling. *Cancer Res*. Apr 1 2011;71(7):2739-49. doi:10.1158/0008-5472.CAN-10-2745

4. Ensign SP, Roos A, Mathews IT*, et al*. SGEF Is Regulated via TWEAK/Fn14/NF-kappaB Signaling and Promotes Survival by Modulation of the DNA Repair Response to Temozolomide. *Mol Cancer Res*. Mar 2016;14(3):302-12. doi:10.1158/1541-7786.MCR-15-0183

5. Komiya Y, Onodera Y, Kuroiwa M*, et al*. The Rho guanine nucleotide exchange factor ARHGEF5 promotes tumor malignancy via epithelial-mesenchymal transition. *Oncogenesis*. Sep 12 2016;5(9):e258. doi:10.1038/oncsis.2016.59

6. Xie C, Zhou X, Liang C*, et al*. Apatinib triggers autophagic and apoptotic cell death via VEGFR2/STAT3/PD-L1 and ROS/Nrf2/p62 signaling in lung cancer. *J Exp Clin Cancer Res*. Aug 24 2021;40(1):266. doi:10.1186/s13046-021-02069-4

7. Cao S, Li L, Geng X, Ma Y, Huang X, Kang X. The upregulation of miR-101 promotes vascular endothelial cell apoptosis and suppresses cell migration in acute coronary syndrome by targeting CDH5. *Int J Clin Exp Pathol*. 2019;12(9):3320-3328.

8. Hashimoto Y, Kurita M, Aiso S, Nishimoto I, Matsuoka M. Humanin inhibits neuronal cell death by interacting with a cytokine receptor complex or complexes involving CNTF receptor alpha/WSX-1/gp130. *Mol Biol Cell*. Jun 2009;20(12):2864-73. doi:10.1091/mbc.E09-02-0168

9. Meucci O, Fatatis A, Simen AA, Miller RJ. Expression of CX3CR1 chemokine receptors on neurons and their role in neuronal survival. *Proc Natl Acad Sci U S A*. Jul 5 2000;97(14):8075-80. doi:10.1073/pnas.090017497

10. Schlegel CR, Fonseca AV, Stocker S*, et al*. DAPK2 is a novel modulator of TRAIL-induced apoptosis. *Cell Death Differ*. Nov 2014;21(11):1780-91. doi:10.1038/cdd.2014.93

11. Jiang Y, Liu J, Xu H, Zhou X, He L, Zhu C. DAPK2 activates NF-kappaB through autophagy-dependent degradation of I-kappaBalpha during thyroid cancer development and progression. *Ann Transl Med*. Jul 2021;9(13):1083. doi:10.21037/atm-21-2062

12. Randall KL, Chan SS, Ma CS*, et al*. DOCK8 deficiency impairs CD8 T cell survival and function in humans and mice. *J Exp Med*. Oct 24 2011;208(11):2305-20. doi:10.1084/jem.20110345

13. Sun Y, Du C, Wang B, Zhang Y, Liu X, Ren G. Up-regulation of eEF1A2 promotes proliferation and inhibits apoptosis in prostate cancer. *Biochem Biophys Res Commun*. Jul 18 2014;450(1):1-6. doi:10.1016/j.bbrc.2014.05.045

14. Alberi L, Sgado P, Simon HH. Engrailed genes are cell-autonomously required to prevent apoptosis in mesencephalic dopaminergic neurons. *Development*. Jul 2004;131(13):3229-36. doi:10.1242/dev.01128

15. Ritch PS, Carroll SL, Sontheimer H. Neuregulin-1 enhances survival of human astrocytic glioma cells. *Glia*. Aug 15 2005;51(3):217-28. doi:10.1002/glia.20197

16. De Bacco F, Orzan F, Erriquez J*, et al*. ERBB3 overexpression due to miR-205 inactivation confers sensitivity to FGF, metabolic activation, and liability to ERBB3 targeting in glioblastoma. *Cell Rep*. Jul 27 2021;36(4):109455. doi:10.1016/j.celrep.2021.109455

17. Cook PJ, Ju BG, Telese F, Wang X, Glass CK, Rosenfeld MG. Tyrosine dephosphorylation of H2AX modulates apoptosis and survival decisions. *Nature*. Apr 2 2009;458(7238):591-6. doi:10.1038/nature07849

18. Chen J, Wang Z, Zheng Z*, et al*. Neuron and microglia/macrophage-derived FGF10 activate neuronal FGFR2/PI3K/Akt signaling and inhibit microglia/macrophages TLR4/NF-kappaB-dependent neuroinflammation to improve functional recovery after spinal cord injury. *Cell Death Dis*. Oct 5 2017;8(10):e3090. doi:10.1038/cddis.2017.490

19. Garces CA, Kurenova EV, Golubovskaya VM, Cance WG. Vascular endothelial growth factor receptor-3 and focal adhesion kinase bind and suppress apoptosis in breast cancer cells. *Cancer Res*. Feb 1 2006;66(3):1446-54. doi:10.1158/0008-5472.CAN-05-1661

20. Meng J, Chen FR, Yan WJ, Lin YK. MiR-15a-5p targets FOSL1 to inhibit proliferation and promote apoptosis of keratinocytes via MAPK/ERK pathway. *J Tissue Viability*. Nov 2021;30(4):544-551. doi:10.1016/j.jtv.2021.08.006

21. Wang D, Bi L, Ran J, Zhang L, Xiao N, Li X. Gas6/Axl signaling pathway promotes proliferation, migration and invasion and inhibits apoptosis in A549 cells. *Exp Ther Med*. Nov 2021;22(5):1321. doi:10.3892/etm.2021.10756

22. Kearon JE, Kocherry SC, Zoumboulakis D, Rivera D, Lourenssen SR, Blennerhassett MG. GDNF requires HIF-1alpha and RET activation for suppression of programmed cell death of enteric neurons by metabolic challenge. *Mol Cell Neurosci*. Sep 2021;115:103655. doi:10.1016/j.mcn.2021.103655

23. Wan YQ, Feng JG, Li M*, et al*. Prefrontal cortex miR-29b-3p plays a key role in the antidepressant-like effect of ketamine in rats. *Exp Mol Med*. Oct 29 2018;50(10):1-14. doi:10.1038/s12276-018-0164-4

24. Edwards HV, Scott JD, Baillie GS. PKA phosphorylation of the small heat-shock protein Hsp20 enhances its cardioprotective effects. *Biochem Soc Trans*. Feb 2012;40(1):210-4. doi:10.1042/BST20110673

25. Zhang B, Zhang HX, Shi ST*, et al*. Interleukin-11 treatment protected against cerebral ischemia/reperfusion injury. *Biomed Pharmacother*. Jul 2019;115:108816. doi:10.1016/j.biopha.2019.108816

26. Dejjuy D, Dechsukhum C, Pattanapanyasat K, Noulsri E, Dissen GA, Leeanansaksiri W. Novel WT1 Target Genes: IL-2, IL-2RB, and IL-2RG Discovered during WT1 Silencing Using Lentiviral-Based RNAi in Myeloid Leukemia Cells. *Biomed Res Int*. 2020;2020:7851414. doi:10.1155/2020/7851414

27. Fu H, Zhang W, Yuan Q*, et al*. PAK1 Promotes the Proliferation and Inhibits Apoptosis of Human Spermatogonial Stem Cells via PDK1/KDR/ZNF367 and ERK1/2 and AKT Pathways. *Mol Ther Nucleic Acids*. Sep 7 2018;12:769-786. doi:10.1016/j.omtn.2018.06.006

28. Lei L, Han D, Gong S, Zheng J, Xu J. Mpz gene suppression by shRNA increases Schwann cell apoptosis in vitro. *Neurol Sci*. Oct 2010;31(5):603-8. doi:10.1007/s10072-010-0341-2

29. Madonna R, Guarnieri S, Kovacshazi C*, et al*. Telomerase/myocardin expressing mesenchymal cells induce survival and cardiovascular markers in cardiac stromal cells undergoing ischaemia/reperfusion. *J Cell Mol Med*. Jun 2021;25(12):5381-5390. doi:10.1111/jcmm.16549

30. Bai Q, Zou M, Zhang J*, et al*. NGF mediates protection of mesenchymal stem cells-conditioned medium against 2,5-hexanedione-induced apoptosis of VSC4.1 cells via Akt/Bad pathway. *Mol Cell Biochem*. Jun 2020;469(1-2):53-64. doi:10.1007/s11010-020-03727-5

31. Halatsch ME, Low S, Mursch K*, et al*. Candidate genes for sensitivity and resistance of human glioblastoma multiforme cell lines to erlotinib. Laboratory investigation. *J Neurosurg*. Aug 2009;111(2):211-8. doi:10.3171/2008.9.JNS08551

32. Mancia Leon WR, Spatazza J, Rakela B*, et al*. Clustered gamma-protocadherins regulate cortical interneuron programmed cell death. *Elife*. Jul 7 2020;9doi:10.7554/eLife.55374

33. Yao X, Li K, Liang C*, et al*. Tectorigenin enhances PDX1 expression and protects pancreatic beta-cells by activating ERK and reducing ER stress. *J Biol Chem*. Sep 11 2020;295(37):12975-12992. doi:10.1074/jbc.RA120.012849

34. Villalba M, Bushway P, Altman A. Protein kinase C-theta mediates a selective T cell survival signal via phosphorylation of BAD. *J Immunol*. May 15 2001;166(10):5955-63. doi:10.4049/jimmunol.166.10.5955

35. Kummer JA, Micheau O, Schneider P*, et al*. Ectopic expression of the serine protease inhibitor PI9 modulates death receptor-mediated apoptosis. *Cell Death Differ*. Aug 2007;14(8):1486-96. doi:10.1038/sj.cdd.4402152

36. Zhang M, Wang B, Ni YH*, et al*. Overexpression of uncoupling protein 4 promotes proliferation and inhibits apoptosis and differentiation of preadipocytes. *Life Sci*. Sep 5 2006;79(15):1428-35. doi:10.1016/j.lfs.2006.04.012

37. Morgan J, Hoekstra AV, Chapman-Davis E, Hardt JL, Kim JJ, Buttin BM. Synuclein-gamma (SNCG) may be a novel prognostic biomarker in uterine papillary serous carcinoma. *Gynecol Oncol*. Aug 2009;114(2):293-8. doi:10.1016/j.ygyno.2009.04.036

38. Sun S, Xue D, Chen Z*, et al*. R406 elicits anti-Warburg effect via Syk-dependent and -independent mechanisms to trigger apoptosis in glioma stem cells. *Cell Death Dis*. May 1 2019;10(5):358. doi:10.1038/s41419-019-1587-0

39. Boutros R, Byrne JA. D53 (TPD52L1) is a cell cycle-regulated protein maximally expressed at the G2-M transition in breast cancer cells. *Exp Cell Res*. Oct 15 2005;310(1):152-65. doi:10.1016/j.yexcr.2005.07.009

40. Lee J, Hoxha E, Song HR. A novel NFIA-NFkappaB feed-forward loop contributes to glioblastoma cell survival. *Neuro Oncol*. Apr 1 2017;19(4):524-534. doi:10.1093/neuonc/now233

41. Tanikawa C, Matsuda K, Fukuda S, Nakamura Y, Arakawa H. p53RDL1 regulates p53-dependent apoptosis. *Nat Cell Biol*. Mar 2003;5(3):216-23. doi:10.1038/ncb943

42. Su F, Overholtzer M, Besser D, Levine AJ. WISP-1 attenuates p53-mediated apoptosis in response to DNA damage through activation of the Akt kinase. *Genes Dev*. Jan 1 2002;16(1):46-57. doi:10.1101/gad.942902

43. Wang W, Guo M, Hu L*, et al*. The zinc finger protein ZNF268 is overexpressed in human cervical cancer and contributes to tumorigenesis via enhancing NF-kappaB signaling. *J Biol Chem*. Dec 14 2012;287(51):42856-66. doi:10.1074/jbc.M112.399923

44. Das S, Raj L, Zhao B*, et al*. Hzf Determines cell survival upon genotoxic stress by modulating p53 transactivation. *Cell*. Aug 24 2007;130(4):624-37. doi:10.1016/j.cell.2007.06.013

45. Pan J, Lian Z, Wallet S, Feitelson MA. The hepatitis B x antigen effector, URG7, blocks tumour necrosis factor alpha-mediated apoptosis by activation of phosphoinositol 3-kinase and beta-catenin. *J Gen Virol*. Dec 2007;88(Pt 12):3275-3285. doi:10.1099/vir.0.83214-0

46. Wang Y, Wu S, Zheng S*, et al*. APOBEC3G acts as a therapeutic target in mesenchymal gliomas by sensitizing cells to radiation-induced cell death. *Oncotarget*. Aug 15 2017;8(33):54285-54296. doi:10.18632/oncotarget.17348

47. He X, Zou H, Wang F. SOX4-induced upregulation of ARHGAP9 promotes the progression of acute myeloid leukemia. *Drug Dev Res*. Dec 2021;82(8):1227-1234. doi:10.1002/ddr.21837

48. Luo J, Liu K, Yao Y*, et al*. DMBX1 promotes tumor proliferation and regulates cell cycle progression via repressing OTX2-mediated transcription of p21 in lung adenocarcinoma cell. *Cancer Lett*. Jul 1 2019;453:45-56. doi:10.1016/j.canlet.2019.03.045

49. Wei H, Hellstrom KE, Hellstrom I. Elafin selectively regulates the sensitivity of ovarian cancer cells to genotoxic drug-induced apoptosis. *Gynecol Oncol*. Jun 2012;125(3):727-33. doi:10.1016/j.ygyno.2012.03.018

50. McGarry N, Greene CM, McElvaney NG, Weldon S, Taggart CC. The Ability of Secretory Leukocyte Protease Inhibitor to Inhibit Apoptosis in Monocytes Is Independent of Its Antiprotease Activity. *J Immunol Res*. 2015;2015:507315. doi:10.1155/2015/507315

51. Deshayes F, Lapree G, Portier A*, et al*. Abnormal production of the TNF-homologue APRIL increases the proliferation of human malignant glioblastoma cell lines via a specific receptor. *Oncogene*. Apr 15 2004;23(17):3005-12. doi:10.1038/sj.onc.1207350

52. Hattermann K, Held-Feindt J, Lucius R*, et al*. The chemokine receptor CXCR7 is highly expressed in human glioma cells and mediates antiapoptotic effects. *Cancer Res*. Apr 15 2010;70(8):3299-308. doi:10.1158/0008-5472.CAN-09-3642

53. Triplett JW, Pavalko FM. Disruption of alpha-actinin-integrin interactions at focal adhesions renders osteoblasts susceptible to apoptosis. *Am J Physiol Cell Physiol*. Nov 2006;291(5):C909-21. doi:10.1152/ajpcell.00113.2006

54. Shioda S, Ohtaki H, Nakamachi T*, et al*. Pleiotropic functions of PACAP in the CNS: neuroprotection and neurodevelopment. *Ann N Y Acad Sci*. Jul 2006;1070:550-60. doi:10.1196/annals.1317.080

55. Kwak HJ, So JN, Lee SJ, Kim I, Koh GY. Angiopoietin-1 is an apoptosis survival factor for endothelial cells. *FEBS Lett*. Apr 9 1999;448(2-3):249-53. doi:10.1016/s0014-5793(99)00378-6

56. Kim I, Kim HG, Kim H*, et al*. Hepatic expression, synthesis and secretion of a novel fibrinogen/angiopoietin-related protein that prevents endothelial-cell apoptosis. *Biochem J*. Mar 15 2000;346 Pt 3:603-10.

57. Fan H, Stefkova J, El-Dahr SS. Susceptibility to metanephric apoptosis in bradykinin B2 receptor null mice via the p53-Bax pathway. *Am J Physiol Renal Physiol*. Sep 2006;291(3):F670-82. doi:10.1152/ajprenal.00037.2006

58. Bramlage CP, Muller GA, Tampe B*, et al*. The role of bone morphogenetic protein-5 (BMP-5) in human nephrosclerosis. *J Nephrol*. Sep-Oct 2011;24(5):647-55. doi:10.5301/JN.2011.6330

59. Li X, Li K, Yu G, Yu C, Liu C. miR-342-5p inhibits expression of Bmp7 to regulate proliferation, differentiation and migration of osteoblasts. *Mol Immunol*. Oct 2019;114:251-259. doi:10.1016/j.molimm.2019.07.027

60. Cheng F, Hu H, Sun K, Yan F, Geng Y. miR-455-3p enhances chondrocytes apoptosis and inflammation by targeting COL2A1 in the in vitro osteoarthritis model. *Biosci Biotechnol Biochem*. Apr 2020;84(4):695-702. doi:10.1080/09168451.2019.1690974

61. Yu ST, Zhong Q, Chen RH*, et al*. CRLF1 promotes malignant phenotypes of papillary thyroid carcinoma by activating the MAPK/ERK and PI3K/AKT pathways. *Cell Death Dis*. Mar 7 2018;9(3):371. doi:10.1038/s41419-018-0352-0

62. Mao YW, Liu JP, Xiang H, Li DW. Human alphaA- and alphaB-crystallins bind to Bax and Bcl-X(S) to sequester their translocation during staurosporine-induced apoptosis. *Cell Death Differ*. May 2004;11(5):512-26. doi:10.1038/sj.cdd.4401384

63. Hu ZX, Geng JM, Liang DM, Luo M, Li ML. Hepatocyte growth factor protects human embryonic stem cell derived-neural progenitors from hydrogen peroxide-induced apoptosis. *Eur J Pharmacol*. Oct 25 2010;645(1-3):23-31. doi:10.1016/j.ejphar.2010.07.011

64. Madge LA, Pober JS. A phosphatidylinositol 3-kinase/Akt pathway, activated by tumor necrosis factor or interleukin-1, inhibits apoptosis but does not activate NFkappaB in human endothelial cells. *J Biol Chem*. May 19 2000;275(20):15458-65. doi:10.1074/jbc.M001237200

65. Ghilardi N, Li J, Hongo JA, Yi S, Gurney A, de Sauvage FJ. A novel type I cytokine receptor is expressed on monocytes, signals proliferation, and activates STAT-3 and STAT-5. *J Biol Chem*. May 10 2002;277(19):16831-6. doi:10.1074/jbc.M201140200

66. Kim MJ, Choi SK, Hong SH*, et al*. Oncogenic IL7R is downregulated by histone deacetylase inhibitor in esophageal squamous cell carcinoma via modulation of acetylated FOXO1. *Int J Oncol*. Jul 2018;53(1):395-403. doi:10.3892/ijo.2018.4392

67. M'Baye M, Hua G, Khan HA, Yang L. RNAi-mediated knockdown of INHBB increases apoptosis and inhibits steroidogenesis in mouse granulosa cells. *J Reprod Dev*. 2015;61(5):391-7. doi:10.1262/jrd.2014-158

68. Saatci O, Kaymak A, Raza U*, et al*. Targeting lysyl oxidase (LOX) overcomes chemotherapy resistance in triple negative breast cancer. *Nat Commun*. May 15 2020;11(1):2416. doi:10.1038/s41467-020-16199-4

69. Saba J, Turati J, Ramirez D*, et al*. Astrocyte truncated tropomyosin receptor kinase B mediates brain-derived neurotrophic factor anti-apoptotic effect leading to neuroprotection. *J Neurochem*. Sep 2018;146(6):686-702. doi:10.1111/jnc.14476

70. Tan W, Dong L, Shi X, Tang Q, Jiang D. P75NTR Exacerbates SCI-induced Mitochondrial Damage and Neuronal Apoptosis Depending on NTRK3. *Curr Neurovasc Res*. 2021;18(5):552-564. doi:10.2174/1567202619666211231091834

71. Kotipatruni RR, Nalla AK, Asuthkar S, Gondi CS, Dinh DH, Rao JS. Apoptosis induced by knockdown of uPAR and MMP-9 is mediated by inactivation of EGFR/STAT3 signaling in medulloblastoma. *PLoS One*. 2012;7(9):e44798. doi:10.1371/journal.pone.0044798

72. Cao F, Xia X, Fan Y*, et al*. Knocking down of Polo-like kinase 2 inhibits cell proliferation and induced cell apoptosis in human glioma cells. *Life Sci*. Apr 1 2021;270:119084. doi:10.1016/j.lfs.2021.119084

73. Tsujii M, DuBois RN. Alterations in cellular adhesion and apoptosis in epithelial cells overexpressing prostaglandin endoperoxide synthase 2. *Cell*. Nov 3 1995;83(3):493-501. doi:10.1016/0092-8674(95)90127-2

74. Wilhelm M, Kukekov NV, Schmit TL*, et al*. Sh3rf2/POSHER protein promotes cell survival by ring-mediated proteasomal degradation of the c-Jun N-terminal kinase scaffold POSH (Plenty of SH3s) protein. *J Biol Chem*. Jan 13 2012;287(3):2247-56. doi:10.1074/jbc.M111.269431

75. Maceyka M, Sankala H, Hait NC*, et al*. SphK1 and SphK2, sphingosine kinase isoenzymes with opposing functions in sphingolipid metabolism. *J Biol Chem*. Nov 4 2005;280(44):37118-29. doi:10.1074/jbc.M502207200

76. Bellail AC, Olson JJ, Yang X, Chen ZJ, Hao C. A20 ubiquitin ligase-mediated polyubiquitination of RIP1 inhibits caspase-8 cleavage and TRAIL-induced apoptosis in glioblastoma. *Cancer Discov*. Feb 2012;2(2):140-55. doi:10.1158/2159-8290.CD-11-0172

77. Salceda S, Tang T, Kmet M*, et al*. The immunomodulatory protein B7-H4 is overexpressed in breast and ovarian cancers and promotes epithelial cell transformation. *Exp Cell Res*. May 15 2005;306(1):128-41. doi:10.1016/j.yexcr.2005.01.018

78. Jia M, Yang X, Yang T*, et al*. beta-1, 3-galactosyltransferase 2 deficiency exacerbates brain injury after transient focal cerebral ischemia in mice. *Brain Res Bull*. Apr 2021;169:104-111. doi:10.1016/j.brainresbull.2021.01.010

79. Koyama-Nasu R, Nasu-Nishimura Y, Todo T*, et al*. The critical role of cyclin D2 in cell cycle progression and tumorigenicity of glioblastoma stem cells. *Oncogene*. Aug 15 2013;32(33):3840-5. doi:10.1038/onc.2012.399

80. Tsuji S, Nakamura S, Yamada T*, et al*. HYBID derived from tumor cells and tumor-associated macrophages contribute to the glioblastoma growth. *Brain Res*. Aug 1 2021;1764:147490. doi:10.1016/j.brainres.2021.147490

81. Liang G, Fang X, Yang Y, Song Y. Silencing of CEMIP suppresses Wnt/beta-catenin/Snail signaling transduction and inhibits EMT program of colorectal cancer cells. *Acta Histochem*. Jan 2018;120(1):56-63. doi:10.1016/j.acthis.2017.11.002

82. Jao TM, Li YL, Lin SW*, et al*. Alteration of colonic epithelial cell differentiation in mice deficient for glucosaminyl N-deacetylase/N-sulfotransferase 4. *Oncotarget*. Dec 20 2016;7(51):84938-84950. doi:10.18632/oncotarget.12915

83. Haas B, Schutte L, Wos-Maganga M, Weickhardt S, Timmer M, Eckstein N. Thioredoxin Confers Intrinsic Resistance to Cytostatic Drugs in Human Glioma Cells. *Int J Mol Sci*. Sep 21 2018;19(10)doi:10.3390/ijms19102874

84. Zhou J, Chng WJ. Roles of thioredoxin binding protein (TXNIP) in oxidative stress, apoptosis and cancer. *Mitochondrion*. May 2013;13(3):163-9. doi:10.1016/j.mito.2012.06.004

85. Wang Z, Boyko T, Tran MC*, et al*. DEL1 protects against chondrocyte apoptosis through integrin binding. *J Surg Res*. Nov 2018;231:1-9. doi:10.1016/j.jss.2018.04.066

86. Feng Y, Tong X, Zhang B, Mao G, Huang H, Ma H. Effect of FAM196B in human lung adenocarcinoma. *J Cancer*. 2018;9(14):2451-2459. doi:10.7150/jca.24907

87. Melino G, Annicchiarico-Petruzzelli M, Piredda L*, et al*. Tissue transglutaminase and apoptosis: sense and antisense transfection studies with human neuroblastoma cells. *Mol Cell Biol*. Oct 1994;14(10):6584-96. doi:10.1128/mcb.14.10.6584-6596.1994

88. Liu Q, Wang Z, Zhou X*, et al*. miR-342-5p inhibits osteosarcoma cell growth, migration, invasion, and sensitivity to Doxorubicin through targeting Wnt7b. *Cell Cycle*. Dec 2019;18(23):3325-3336. doi:10.1080/15384101.2019.1676087
